# Epidemiology and Genetic Variability of Circulating Influenza B Viruses in Uruguay, 2012–2019

**DOI:** 10.3390/microorganisms8040591

**Published:** 2020-04-19

**Authors:** María José Rivas, Miguel Alegretti, Leticia Cóppola, Viviana Ramas, Héctor Chiparelli, Natalia Goñi

**Affiliations:** 1 Centro Nacional de Referencia de Influenza, Unidad de Virología, Departamento de Laboratorios de Salud Pública, Ministerio de Salud, Montevideo 11600, Uruguay; marjosrivas@gmail.com (M.J.R.); lcoppola@msp.gub.uy (L.C.); vramas@msp.gub.uy (V.R.); hchiparelli@msp.gub.uy (H.C.); 2 Departamento de Vigilancia en Salud, Ministerio de Salud, Montevideo 11200, Uruguay; malegretti@msp.gub.uy

**Keywords:** influenza B, phylogenetic, epidemiology, surveillance, victoria, yamagata

## Abstract

Influenza B viruses (IBV) are an important cause of morbidity and mortality during interpandemic periods in the human population. Two phylogenetically distinct IBV lineages, B/Yamagata and B/Victoria, co-circulate worldwide and they present challenges for vaccine strain selection. Until the present study, there was little information regarding the pattern of the circulating strains of IBV in Uruguay. A subset of positive influenza B samples from influenza-like illness (ILI) outpatients and severe acute respiratory illness (SARI) inpatients detected in sentinel hospitals in Uruguay during 2012–2019 were selected. The sequencing of the hemagglutinin (HA) and neuraminidase (NA) genes showed substitutions at the amino acid level. Phylogenetic analysis reveals the co-circulation of both lineages in almost all seasonal epidemics in Uruguay, and allows recognizing a lineage-level vaccine mismatch in approximately one-third of the seasons studied. The epidemiological results show that the proportion of IBV found in ILI was significantly higher than the observed in SARI cases across different groups of age (9.7% ILI, 3.2% SARI) and patients between 5–14 years constituted the majority (33%) of all influenza B infection (*p* < 0.05). Interestingly, we found that individuals >25 years were particularly vulnerable to Yamagata lineage infections.

## 1. Introduction

Influenza viruses are considered to be a major human health problem with a global distribution, with considerable impact on the quality of life and productivity of the society. An estimated of more than 290,000 seasonal influenza-associated respiratory deaths annually occur worldwide [1]. In the American region, each year, an average of 772,000 respiratory hospitalizations is attributed to influenza virus [2]. Although influenza A viruses (IAV) are responsible for pandemics and they have major rates of hospitalization and mortality attribution, influenza B viruses (IBV) cause epidemics worldwide annually, becoming responsible in some years for more than 25% of human seasonal influenza infections, and its prevention represents an important public health priority [3,4,5]. In vaccine mismatch seasons, hospitalization due to IBV can exceed IAV in all age groups, particularly among children and young teenagers [6].

IBV were first identified in the 1940s and, unlike influenza A, they primarily cause disease in humans [7]. Recurrent seasonal epidemics are the consequence of the antigenic drift, ongoing changes in the amino-acid sequences at antigenic sites in the two surface glycoproteins hemagglutinin (HA) and neuraminidase (NA) [8]. During the early 1980s, evolutionary changes in the HA gene diverged into two phylogenetically and antigenically distinct lineages that are represented by B/Victoria/2/87 (Victoria lineage) and B/Yamagata/16/88 (Yamagata lineage) [9]. Since 2001, both lineages have co-circulated, showing distinct antigenicity and transmission dynamics and often alternating in regional dominance presenting challenges for vaccine strain selection [10]. Importantly, the co-circulation of the two lineages results in a different pattern of evolution of influenza B virus and can explain some of the disparate variability of seasonal outbreaks [11]. Recent reports have highlighted the potential differences in the epidemiology of Victoria and Yamagata lineage viruses, including younger average ages of persons with Victoria virus infection and greater transmissibility. The Victoria lineage is under greater positive selection pressure and, hence, likely to experience greater antigenic drift than the more conserved Yamagata lineage [12]. Annual influenza vaccination is the most effective method for preventing influenza and its associated complications. In general, seasonal vaccines are trivalent, including one influenza B lineage. Despite being only two lineages, the selection of the right influenza B virus strain in the vaccine has been proven very difficult, because the dominant lineage changes over time, as result, the vaccine efficacy decreased when the included vaccine strain did not match the circulating epidemic strain [13].

The aim of this study was to describe the circulating patterns of IBV strains in Uruguay between 2012–2019 as well as determine the age groups that are associated with both influenza B lineages and surveillance type. In addition, we evaluated the concordance between the predominant circulating influenza B lineage and the B lineage included in the vaccine applied in each season. Additionally, we intended to determine the phylogenetic relationships between the circulating strains in Uruguay and neighbouring countries, and the vaccine strains recommended for the Southern Hemisphere. For this purpose, we firstly analyzed the genetic sequences of the HA and NA genes that were obtained in this study and compared with sequences downloaded from the Global Initiative on Sharing all Influenza data (GISAID) database. Further, the co-circulation of IAV and IBV were examined. Finally, we analyzed the NA gene to observe the existence of possible known substitutions that cause reduced susceptibility to neuraminidase inhibitors (NAI).

## 2. Materials and Methods

### 2.1. Influenza Surveillance and Sample Collection

The National Influenza Center (NIC) located in Montevideo, Uruguay, in the Virology Unit of the Department of Public Health Laboratories, Ministry of Health, is the Reference Laboratory for Influenza and other non-Influenza respiratory viruses. Uruguay is a country with a temperate climate, where outbreaks of respiratory viruses usually occur mainly in the autumn-winter period, between April and October. The virological surveillance system is based on the year-round sampling effort and it was carried out in six sentinel centers, including five general hospitals and one children´s hospital strategically located in different regions of the country. Nasopharyngeal aspirates or nasal swabs that were obtained from 5299 hospitalized patients with severe acute respiratory infection (SARI) associated with influenza and 455 outpatients with clinical evidence of influenza-like illness (ILI) were collected between 2012 and 2019. The ILI surveillance was implemented in 2017. All six ILI sentinel centers were the same from the existing SARI surveillance network.

### 2.2. Ethical Considerations

As a laboratory within the World Health Organization (WHO) Global Influenza Surveillance and Response System (GISRS) for the purposes of global surveillance of influenza under the WHO Global Influenza Program, neither written informed consent nor explicit ethical approval were sought, as this study was only observational and carried out as part of a routine virological surveillance (anonymously, without identification of patients), as established in the terms of reference for WHO National Influenza Centers.

### 2.3. Statistical Methods 

The Chi square test was applied in order to evaluate the differences in percent positivity between the compared groups. A *p*-value < 0.05 was considered statistically significant. 

The proportion of influenza B positives among influenza virus positive specimens was separately determined in all years for all surveillance programs. The proportion of B/Victoria and B/Yamagata circulating in each year was also determined among specimens that could be typed.

Comparisons of influenza B lineages distribution by age were completed by the Pearson Chi square test and median test. A *p*-value <0.05 was considered to be statistically significant. In addition, we examined the proportion of each influenza B lineage across different age groups in the study. All analyses were performed using Epidat (version 4.1), STATA and Excel software. 

### 2.4. Molecular Detection of Influenza B Viruses

#### 2.4.1. Extraction and Real Time RT-PCR Assays for Differentiation of Influenza B Lineages

Ribonucleic acid (RNA) was extracted from 140 μL of clinical samples that were stored at −80 °C, using QIAamp Viral RNA mini kit (Qiagen), according to the manufacturer´s suggested protocol.The influenza B positive specimens previously typified were distinguished into influenza B Victoria and Yamagata lineages by RT-PCR assay. Real time RT-PCR was performed by using a Invitrogen SuperScript™ III Platinum™ One-Step Quantitative RT-PCR System according to the manufacturer´s instructions, on an Applied Biosystems™Step One Plus (Applied Biosystems, USA) thermocycler. Influenza B lineage-specific sets of primers and probes were provided by Centers for Disease Control and Prevention (CDC, USA). Testing was done according to the CDC´s instructions, being enclosed with reagents. In brief, the master mix containing appropiate primers and probe was dispensed into reaction tube plate in a volume of 20 μL, and 5 μL of RNA extracts were added to each well. Negative and positive template controls for all of the primer/probe sets were included in each run. The human Rnase P gene primer and probe set served as an internal positive control for human RNA. Thermocycling real time RT-PCR conditions were as follows: reverse transcription for 30 min at 50 °C, Taq polymerase activation for 10 min at 95 °C, and 45 cycles of denaturation at 95 °C for 15 s, and extension at 55 °C for 30 s. The results were analyzed using Applied Biosystems Step OnePlus Software 2.1 version and the interpretation of the data was done according to the WHO guidelines [14].

#### 2.4.2. PCR and Sequencing

For the HA gene, the amplification of a region that contains the HA1-coding region (1340 base pairs) was performed while using the SS III One-Step RT-PCR System with Platinum™ Taq HiFi Kit and the RT-PCR assays with primer sets that were recommended by the World Health Organization (WHO) [15]. Briefly, the 50 μL reaction volume contained 25 μL of 2× PCR buffer, 15.5 μL of RNAse-free H_2_O, 1.5 μL of each 10 μM reverse and forward primer, 1 μL enzyme mix (Taq DNA polymerase and reverse transcriptase), 0.5 μL of RNAsin, and 5 μL of viral RNA extract. The RT-PCR reaction was carried out in a Biorad T100 thermocycler with a single reverse transcription step of 42 °C for 30 min, 50 °C 10 min, a pre-denaturation (94 °C) for 2 min, forty cycles (denaturation 30 s at 94 °C, 30 s. of primer annealing al 55 °C, 2 min 30 s of extension al 68 °C), and a final elongation step of 10 min at 68 °C. For the NA gene, a specific primer set was designed for the partial amplification of the NA gene (position 168−1386 bp). Briefly, the 50 μL reaction volume contained 25 μL of 2× PCRbuffer, 17 μL of RNAse-free H2O, 1 μL of each 10uM reverse (5′AACACCTGTGACAGTGTCCC3′) and forward primer (5′CGCATCAAATGTTCAGGCTG3′), 1 μL enzyme mix (Taq DNA polymerase and reverse transcriptase), and 5 μL of viral RNA extract. The RT-PCR reaction was carried out in a Biometra thermocycler with a single reverse transcription step of 50 °C for 30 min, “hot start PCR” (94 °C) for 2 min, thirty five cycles (denaturation 1 min at 94 °C, 1 min of primer annealing at 54 °C, 2 min of extension at 72 °C), and a final elongation step of 10 min at 72°C. RT-PCR products from the HA and NA genes were separated in a 1% agarose gel with GelRed™ (Biotium, Hayward, CA, USA) and then visualized under ultraviolet light. The amplified products were purified with Pure link™ (Invitrogen), according to the manufacturer’s directions, and sequencing was performed at Macrogen, Korea and the Instituto Pasteur in Montevideo, Uruguay. 

### 2.5. Sequence Data

In addition to the 39 HA and 27 NA sequences that were obtained in this study, other sequences from Uruguay and the neighboring countries available at the GISAID database from 2012–2019 were downloaded and included in the phylogenetic analysis as well as those vaccine strains for the Southern Hemisphere that were recommended by WHO between 2012–2019 [16]. The output was a dataset containing around a hundred sequences. HA and NA nucleotide sequences of the Uruguayan influenza B viruses generated in this study have been deposited in the GISAID database. For names and accession numbers, see Supplementary Material Table S1. The sequences were assembled while using Lasergene analysis software, version 10 (DNASTAR, Inc., Madison, WI, USA) and manually edited to produce a final sequence of 1201 nucleotides (nt) for the HA1 region of the HA gene and 1108 nt for the NA gene.

### 2.6. Phylogenetic Analysis 

Multiple sequence alignment was performed by using the Muscle method implemented in Mega program (v 6.06) (http://www.megasoftware.net/) [17]. The phylogenetic trees were constructed applying the neighbor-joining method with Kimura´s two-parameter distance model [18,19]. For each tree, the reliability of phylogenetic groupings was determined through a bootstrap analysis with 1000 replicates. Only bootstrap values above 70% were identified as distinct groups [20]. The trees were visualized using Fig Tree version 1.4.2 (http://tree.bio.ed.ac.uk/software/figtree). 

## 3. Results

### 3.1. Age Distribution among Type of Surveillance

From 2012 to 2019, a total of 5299 SARI and 455 ILI cases were reported. Age distributions show that children aged 0–4 years old were the largest group of ILI (166/455, 36%) and SARI (3044/5299, 57%) patients (Table 1). The median age of the tested patients was four years and the median age of ILI patients (10 years) was significantly older than those of SARI patients (*p* < 0.05). 

### 3.2. Prevalence of Influenza B Viruses

**Figure 1:**
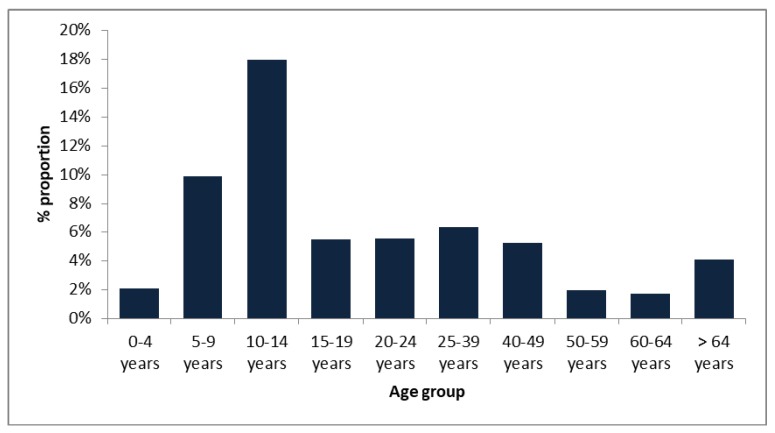
Distribution of influenza B viruses (IBV) cases in Uruguay according to age groups, 2012–2019.The proportion of positive samples for IBV for the different age groups are indicated.

In the eight-year period studied, the NIC received a total of 5754 samples from the sentinel surveillance. Of these specimens, 217 (3.8%) were confirmed for influenza B virus by real-time RT-PCR. During the entire study period, Victoria lineage viruses were more represented than those that belonged to the Yamagata lineage, accounting for 51% (110/217) and 45% (98/217) of cases, respectively. The remaining 4 % (9/217) could not be attributed to a specific lineage. The 217 IBV ranged from newborn to 88-year old. Influenza B infection was widely distributed across age-groups, with a clear predominance in children and teenagers with more than 60% of total influenza B infections. Those between 5–14 years had the highest detection rates among all age groups for both lineages (Figure 1).

The mean and median ages for the IBV detected were 22 and eight years, respectively. For ILI patients, the highest rate is in the 5–9 years group (29.5%, 13/44), followed by children <4 years (20.4%, 9/44) and 10–14 years 18.2%, 8/44). We observed that, as the age increased, the circulation of the virus decreased becoming practically null in ages > 39 years. Meanwhile, for SARI patients, we observed two main circulation peaks being the positive rate of influenza B viruses of SARI patients highest in children <4 years (33%, 57/172), followed by >64 years (19%, 33/172). Overall, and even though ILI surveillance was only done during three of the eight years of the study, the proportion of influenza B found was significantly higher than that observed among SARI cases across different groups of age (9.7%, 44/450 ILI and 3.2%, 172/5299 SARI, *p* < 0.05).

Some differences were observed regarding the circulation of both lineages according to age. Although the proportions of cases fewer than 4 years were similar in both lineages (18%, 39/208 B/Victoria, 13% 27/208 B/Yamagata), patients aged between 5–14 years were most frequently infected by B/Victoria lineage (22% 46/208 B/Victoria, 11% 23/208 B/Yamagata). However, in ages >24 years B/Yamagata viruses circulated in a higher proportion (21% 45/208 B/Yamagata, 10% 21/208 B/Victoria) (Table 1).

### 3.3. Interactions among Influenza A and B Viruses

**Figure 2:**
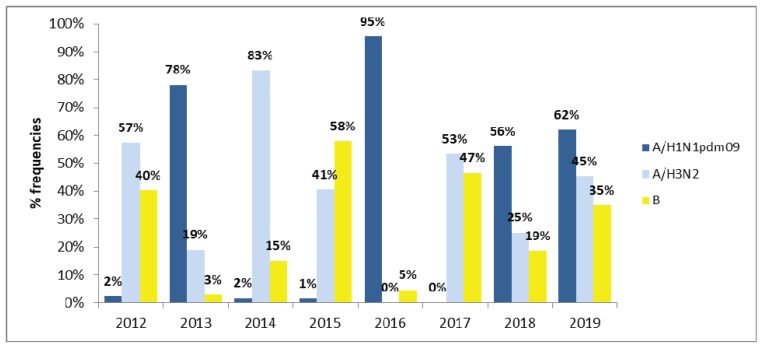
Incidence of influenza A and B viruses identified from clinical samples in Uruguay during 2012 to 2019. The year distribution of the influenza viruses, including A (H1N1pdm09) (blue color), A(H3N2) (light blue) and influenza B (yellow color) activity.

Fluctuations in the prevalence of influenza A and B were observed during 2012–2019. Overall, 14.6% were confirmed influenza cases, of which 36.9% (311/843), 37.4 % (315/843), and 25.7% (217/843) were influenza A/H1N1 pdm09, A/H3N2, and B infections, respectively. Influenza B viruses rarely dominate over influenza A viruses, however in 2012, 2015, and 2017 there was an important circulation of influenza B viruses, reaching 40%, 58% and 47%, respectively, of the circulating influenza virus. The co-circulation of influenza A/H3N2 and B was observed in almost all of the periods studied (2012, 2013, 2014, 2015, 2017, and 2018). However, in 2013 and 2016, the lowest incidence of influenza B virus infection was detected (2.8% and 4.5%, respectively), along with the highest incidence of influenza A/H1N1 pdm09 (78% and 95%, respectively) (Figure 2). 

### 3.4. Mismatches between Circulating Strains of Influenza B and Vaccine Strains 

**Figure 3:**
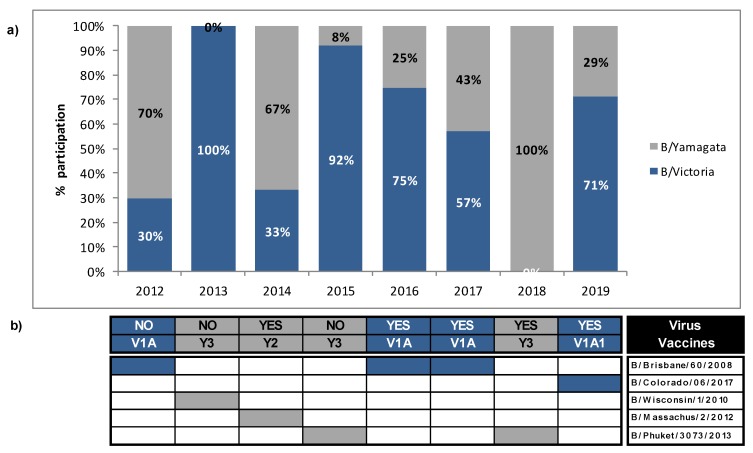
Circulation of influenza B lineages according to season (year) and recommended vaccine lineage, Uruguay, 2012 to 2019. (**a**) Circulation percentages of both lineages for each season based on samples that could be subtyped. Blue boxes represent B/Victoria lineage; grey boxes represent B/Yamagata lineage. (**b**) Vaccine strains used for each year are indicated, and “Yes” or “No” if they coincide with the influenza B strains that have circulated. Abbreviations V1A, V1A1, Y2, and Y3 correspond to clade 1A of B/Victoria lineage, subclade 1 of B/Victoria clade 1 A lineage, clade 2 of B/Yamagata lineage, and clade 3 of B/Yamagata lineage, respectively.

Influenza B lineage mismatch was defined as a season when >60% of circulating B lineage virus was different to the lineage that was included in the trivalent influenza vaccine (TIV) for that season. A partial mismatch was defined if both lineages co-circulated at equal or almost equal proportions (40–59%). During 2012–2019, Victoria and Yamagata-lineage co-circulated in Uruguay, most of the years, with alternated predominance. However, only one lineage is contained in the trivalent vaccine recommended by the WHO for each season. We evaluated the extent of lineage-level mismatch between the circulating B viruses and those that were included in vaccine formulation for the Southern Hemisphere. The proportion of circulating IBV mismatched to the vaccine strain was calculated based in the total number of successfully lineage-typed specimens. During 2012–2019, a high degree of B mismatch in the 2012, 2013, and 2015 seasons (70%, 100%, and 92%, respectively) were observed. It is noteworthy that, in 2013, IBV circulation was very low and only B/Victoria strains were detected. On average, 28% of circulating influenza B was mismatched to recommended influenza B vaccine strain during 2012, 2013, and 2015. During 2017, a partial mismatch (Victoria 57%, Yamagata 43%) was observed, being the vaccine strain from the Victoria lineage. Meanwhile, in the 2014, 2016, 2018, and 2019 seasons, the lineage of the dominant sequences matched the lineage of the vaccine strain that was selected in the same season (Figure 3).

### 3.5. Molecular Detection of Influenza B Subtypes

Subtyping real-time RT-PCR revealed that 110 samples were B/Victoria and 98 were B/Yamagata lineage, respectively, to identify the lineage of the 217 influenza B viruses circulating in Uruguay during 2012–2019. The remaining nine samples could not be subtyped. Forty-one samples were randomly selected from 208 influenza B virus for further HA and NA genetic analyses.

#### 3.5.1. Sequence and Phylogenetic Analysis of the HA Gene of Uruguayan Influenza B Viruses

**Figure 4:**
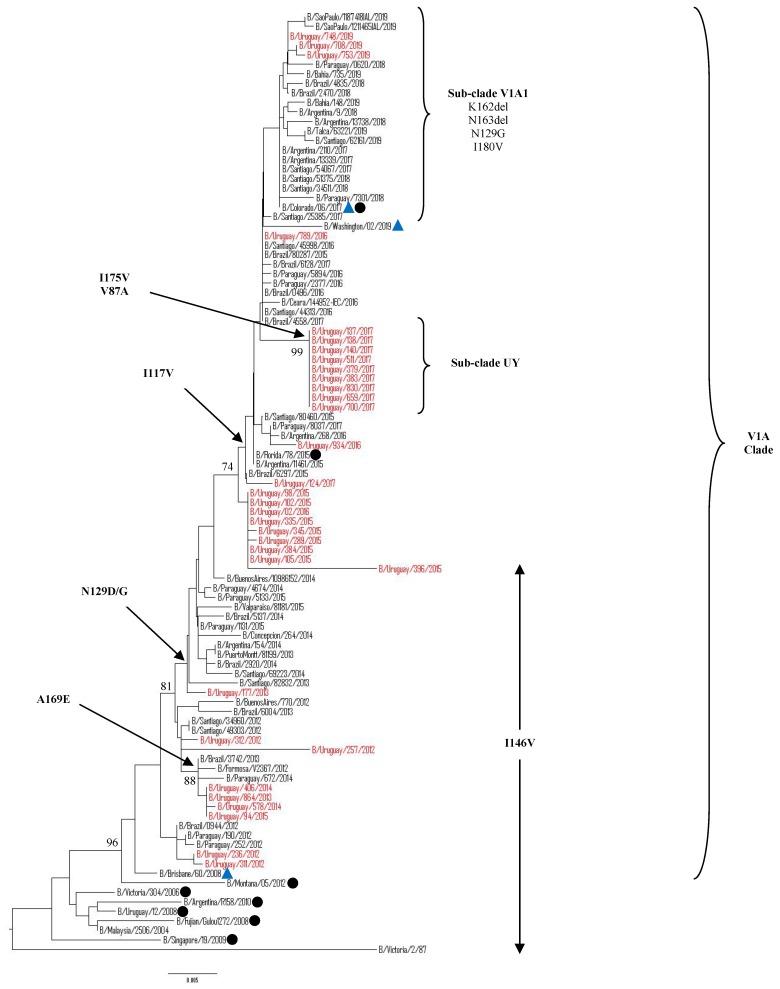
Phylogenetic analysis of the HA1 nucleotide sequences from influenza B Victoria-lineage viruses circulating in Uruguay from 2012 to 2019. The phylogeny tree was generated by the neighbor-joining method with 1000 bootstrap replicates. Bootstrap values >70 are shown in the nodes. Vaccine strains are preceded by blue triangles. Uruguayan strains are shown in red while representative clade strains are indicated with a black circle as a symbol. The scale bar indicates nucleotide substitutions per site. The most relevant amino acid substitutions found are indicated with arrows.

**Figure 5:**
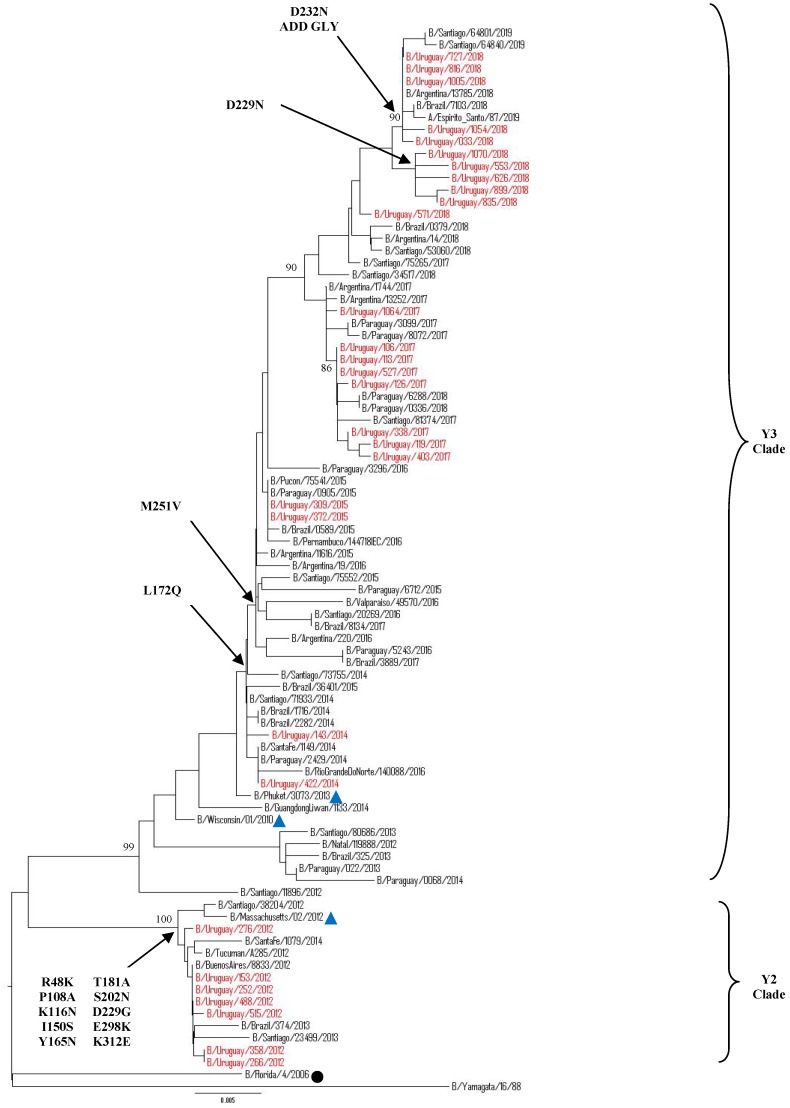
Phylogenetic analysis of HA1 nucleotide sequences from influenza B Yamagata-lineage viruses circulating in Uruguay from 2012 to 2019. The phylogeny tree was generated by the neighbour-joining method with1000 bootstrap replicates. Bootstrap values >70 are shown in the nodes. Vaccine strains are shown with a blue triangle. Uruguayan strains are shown in red while representative clade strains are indicated with a black circle as a symbol. The scale bar indicates the substitutions per site. The most relevant amino acid substitutions found are indicated with arrows.

The complete HA1 region of the HA gene were obtained from 39/41 of the selected samples. Additional 24 Uruguayan HA influenza B sequences were obtained from GISAID database. Hence, a total of 63 HA influenza B sequences from 208 positive samples from 2012 to 2019 were included for phylogenetic analysis. Among 63 sequences, 33 belonged to Victoria lineage and 30 belonged to Yamagata lineage. The phylogenetic trees showed different genetic diversities of both lineages based on nucleotide differences in the HA1 region. B/Victoria and B/Yamagata lineages co-circulated in almost all seasonal epidemics, as shown in Figure 4 and 5. In 2013, only the Victoria lineage was detected whereas in 2018 only Yamagata lineage was detected in Uruguayan samples. Although co-circulation of Victoria and Yamagata lineages was observed, the Victoria lineage clade V1A predominated during 2013, 2015–2017, in 2018 there was no Victoria viruses circulating and reappeared in 2019 assubclade V1A.1, while the Yamagata-lineage strains predominated in 2012 as clade 2 and clade 3 in the 2014 and 2018 seasons.

Victoria Lineage 

Our 2012–2017 Uruguayan influenza B/Victoria HA sequences were grouped in clade 1A, together with the vaccine strain B/Brisbane/60/2008, as recommended by the WHO to be used in 2012, 2016, and 2017 seasons. It is noteworthy that, in 2018, there was no detection of B/Victoria lineage strains in Uruguay, unlike what happened in neighboring countries. The HA gene sequences from 2019 Uruguayan circulating viruses were grouped in subclade V1A.1, within clade 1 A along with 2017, 2018, and 2019 HA sequences from neighboring countries. These subclade viruses were closely related to B/Colorado/60/2017, the vaccine strain recommendation by the WHO to be used in 2019 in the Southern Hemisphere. When comparing to vaccine strain B/Brisbane/60/2008, a series of amino acid changes were observed in the HA1 region. All of the Uruguayan sequences in the 2012–2014 seasons shared amino acid substitutions at I146V, whereas the 2015–2017 seasons shared amino acids substitutions at I117V and N129D. Interestingly, nine of 10 Uruguayan 2017 sequences shared additional amino acids substitutions V87A and I175V only found in Uruguayan sequences. Although these sequences do not have other substitutions within this cluster that differentiates one from another, the epidemiological information shows different geographic locations and different sample dates for seven of them. All of the 2019 Uruguayan sequences were characterized by a 2-aa deletion (K162 and N163) in the HA protein. These viruses had additional substitutions of N129G, I180V (Figure 4).

Yamagata Lineage 

Our 2012–2019 Uruguayan influenza B/Yamagata HA sequences clustered into two distinguishable genetic clades: clade 2 and 3. All B/Yamagata HA sequences detected after 2013 belonged to clade 3, closely related to the vaccine strains B/Wisconsin/01/2010 (in 2013 season) and B/Phuket/3073/2013 (in 2015 and 2018 seasons), whereas only HA sequences from 2012 belonged to clade 2 and were related to vaccine strain B/Massachusetts/02/2012, the vaccine strain recommendation for WHO to be used in 2014 in the Southern Hemisphere. When comparing with the vaccine strain B/Phuket/3073/2013, a series of amino acid changes were observed in the HA1 region. All of the Uruguayan sequences in the 2012 season shared amino acid substitutions at P108A, K116N, R48K, I150S, Y165N, T181A, S202N, D229G, E298K and K312Ewith some having additional substitutions. All of the Uruguayan Yamagata lineage sequences from the 2014–2017 seasons shared amino acids substitutions L172Q and M251V. In 2018, some subclustering of sequences, as defined by specific amino acid substitutions (e.g., D229Nor D232N [introducing a potential N-linked glycosylation site]), can be seen amongst the most recently circulating viruses characterized (Figure 5). Interestingly, Uruguayan sequences that were grouped in the D232N subclade belonged from outpatients samples.

Between 2012–2014, we observed a clade shift inside the Yamagata lineage: in 2012, all Uruguayan sequences were clade 2, in 2013 there was no Yamagata viruses circulating in Uruguay, re-appearing in 2014 within clade 3. It was observed in countries of the region, not in Uruguay, that during 2012–2014 there were different clades co-circulating within the same period.

Mutations in the HA1 domain

Previous structural studies of the HA1subunit of HA have identified four major epitopes comprising the 120-loop (position 116–137 of HA1), 150-loop (position 141–150), 160-loop (position 162–167), and 190-helix (position 194–202), which collectively form part of the receptor binding site [21]. We detected many amino acid substitutions when compared to respective representative candidate vaccine strains, as shown in Tables S2 and S3. Relative to B/Brisbane/60/2008, there were 13 mutation sites in the HA1 region in our B/Victoria samples. The most represented substitutions were I117V, N129D in the 120-loop, and I146V in the 150-loop. Interestingly, two amino acid substitutions located near antigenic epitopes, V87A and I175V, have been found in 2017 Uruguayan sequences. Unexpectedly, these substitutions have not been found in all other sequences from neighboring countries and included in these studies. We performed the same analysis in the Yamagata lineage. Relative to B/Phuket/3073/2013, there were 15 mutation sites in the HA1 region of our B/Yamagata samples. The most represented substitutions were L172Q, D229N/G, D232N, and M251V, being located near the antigenic epitopes as well as a few mutations in the antigenic epitopes: K116N in the 120-loop area, I150S in the 150-loop area, Y165N in the 160 loop-area, and S202N in the 190-helix area.

#### 3.5.2. Sequence and Phylogenetic Analysis of the NA Gene of Uruguayan Influenza B Viruses

**Figure 6:**
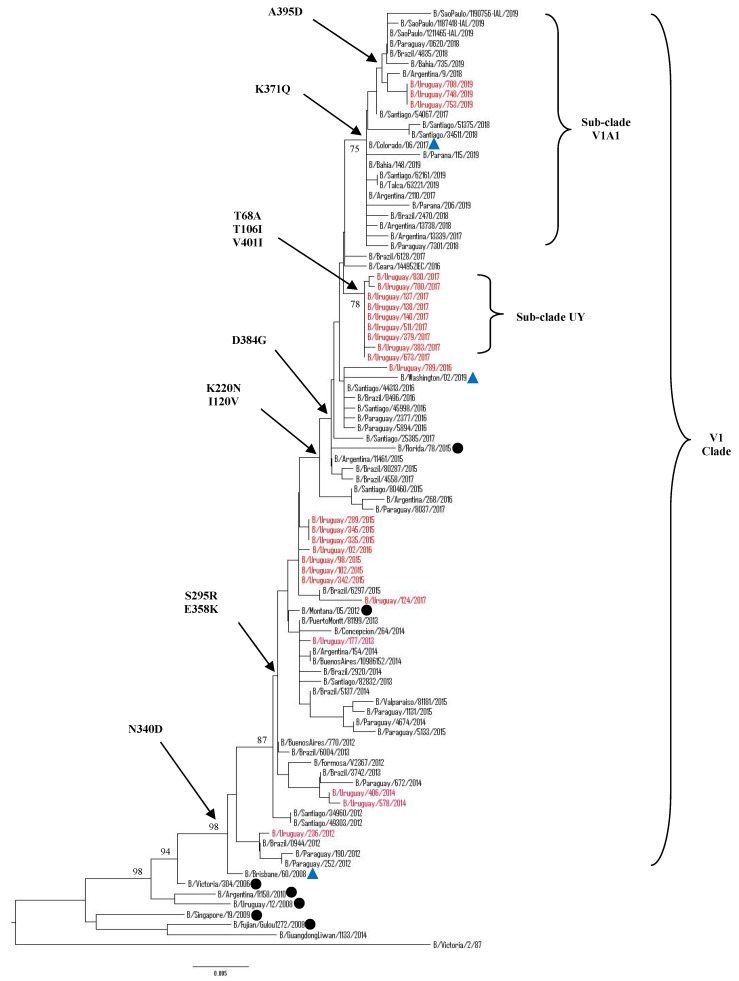
Phylogenetic analysis of the partial neuraminidase nucleotide sequences from influenza B Victoria-lineage viruses circulating in Uruguay from 2012 to 2019.The phylogeny tree was generated by the neighbour-joining method with 1000 bootstrap replicates. Bootstrap values >70 are shown in the nodes. Vaccine strains are shown with a blue triangle. Uruguayan strains are shown in red while representative clade strains are indicated with a black circle as a symbol. The scale bar indicates substitutions per site. The most relevant amino acid substitutions found are indicated with arrows.

A partial region of the NA gene was obtained from 27/41 of the selected samples. An additional 24 Uruguayan NA influenza B sequences were obtained from GISAID database. Hence, a total of 51 NA influenza B sequences from 208 positive samples from 2012 to 2019 were included for phylogenetic classification. Among the 51 sequences, 25 belonged to Victoria lineage and 26 belonged to Yamagata lineage. All of the Uruguayan NA sequences of Victoria lineage were classified into clade 1 (Figure 6). Comparing to strain B/Brisbane/60/2008, N340D amino acid substitution was found in all seasons. Since 2013, S295R, E358K amino acid substitutions were circulating in Uruguayan viruses, then strains of 2016 season and on, showed amino acids substitutions I120V, K220N and D384G. The 2017 Uruguayan NA sequences showed special and unique amino acids substitutions T68A, T106I, and V401I, being the same samples that had unique substitutions V87A and I175V in the HA gene.

The 2019 Uruguayan NA gene phylogeny were largely congruent with the HA phylogeny and the 162–163 group of viruses are defined by NA K371Q amino acid substitution (Table S4).

**Figure 7:**
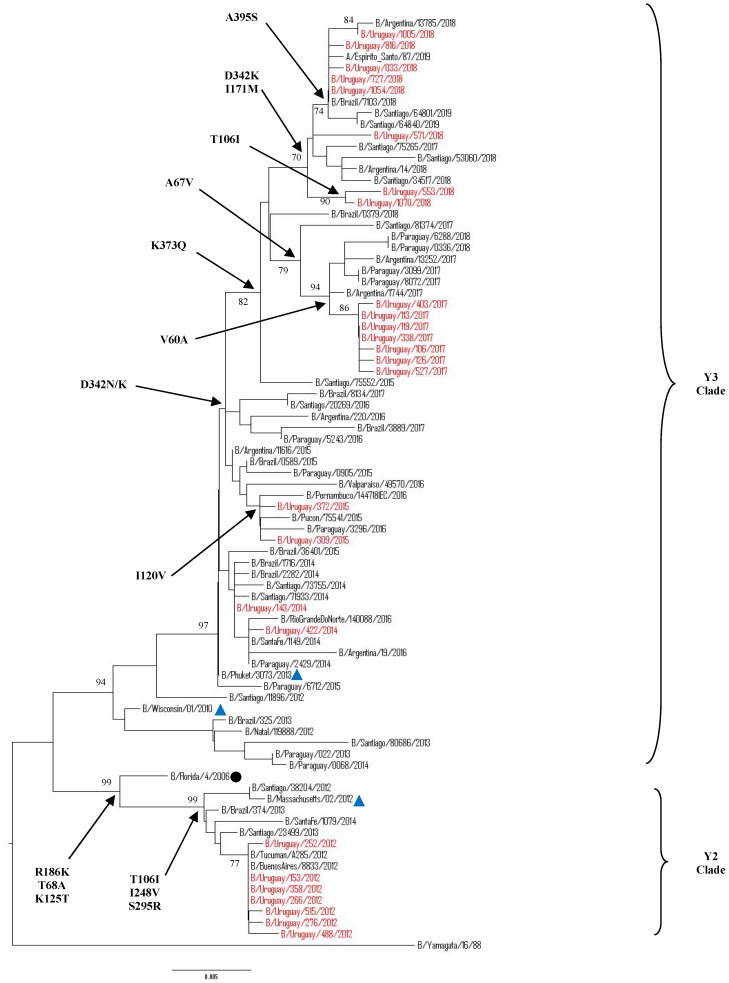
Phylogenetic analysis of the partial neuraminidase nucleotide sequences from influenza B Yamagata-lineage viruses circulating in Uruguay from 2012 to 2019. The phylogeny tree was generated by the neighbor-joining method with 1000 bootstrap replicates. Bootstrap values >70 are shown in the nodes. Vaccine strains are shown with a blue triangle. Uruguayan strains are shown in red while representative clade strains are indicated with a black circle as a symbol. The scale bar indicates substitutions per site. The most relevant amino acid substitutions found are indicated with arrows.

Our 2012–2019 Uruguayan influenza Yamagata-lineage NA sequences clustered into two distinguishable genetic clades: clade 2 and 3 (Figure 7). Almost all of the B sequences detected after 2013 were classified as clade 3 closely related to B/Phuket/3073/2013, whereas only NA sequences from 2012 fell into clade 2 and related to strain B/Massachusetts/02/2012. When comparing to strain B/Phuket/3073/2013, a series of amino acid changes was observed in clade 2 which included B/Florida/4/2006, and was characterized by T106I and S295R substitutions, while clade 3, which included B/Wisconsin/1/2010, was distinguished by K373Q, D342N, and I171M. Interestingly, after 2017, in Uruguay and the region the substitution in position D342N changed to D342K. The 2018 Uruguayan NA sequences showed an amino acid substitution T106I, being the same samples that had unique substitution D229N in the HA gene (Table S5).

There are several important conserved residues in the NA active site of influenza B virus [22]. The NA protein of influenza B has eight catalytic residues (R116, D149, R150, R223, E275, R292, R374, and Y409) that interact with sialic acid and 11 framework residues (E117, R154, W177, S178, D197, I221, E226, H273, E276, N293, and E248) that support the enzymatic binding pocket [23]. None of the 51 NA protein sequences that we analyzed displayed substitutions in the active site and their surrounding residues.

We checked whether the Uruguayan sequences analyzed for the NA gene were classified in the same lineages according to HA gene due to the possibility that influenza viruses undergo gene reassortment. Although the classification of the two lineages is based on HA sequences, it is clear that most NA sequences are also defined under specific lineage clades with their lineage identified according to the vaccine strains clustered with them. No occurrence of HA and NA reassortment events were observed in the Uruguayan sequences during 2012–2019 (Figure 6 and Figure 7).

Mutation Screening for Influenza B Virus Resistance to NA Inhibitors

Influenza NA is a surface glycoprotein that enables the virus to be released from the infected host cells. Using inhibitors to block the cleavage function of influenza NA does not avoid disease, but it treats influenza disease and might shorten the symptoms. We analyzed the NA gene of 51 samples for specific mutations known to confer resistance to NA inhibitors [24,25]. None of the Uruguayan NA sequences from original clinical samples had substitutions for influenza B virus resistance to NA inhibitors, which suggested that the antivirals in use might still be suitable as a first-line treatment of influenza B infections in Uruguay.

## 4. Discussion

Until the present study, there was little information regarding the epidemiology and genetic characteristics of the circulating influenza B viruses in Uruguay [26,27]. On average, influenza B viruses accounted for approximately 26% of influenza positive cases during 2012–2019. These results were similar to other countries that suggest that influenza B virus contribute significantly to influenza disease [3,28,29,30,31,32,33]. Our findings revealed that types A and B influenza viruses almost always co-circulated throughout 2012–2019 and they confirm the important role of influenza B virus in the spread of infection in the population. In a study by Glezen P and colleagues [4], it was suggested that when influenza B activity is intense, it can produce an impact that is similar to that of influenza A. Influenza B viruses rarely dominate in a season, however in 2015, in Uruguay, predominated over influenza A. The increase of influenza B incidence also coincided with a shift in the predominant influenza B lineage (from the Yamagata lineage in 2014 to Victoria lineage in 2015) and clade (from Yamagata clade 2 in 2012 to Yamagata clade 3 in 2014). In 2013 and 2016, the lowest incidence of influenza B virus infection was detected in Uruguay, along with the highest incidence of influenza A/H1N1 pdm09. The absence of influenza B viruses in some season is not unusual, since it is known that the activity of the influenza B virus is highly variable worldwide, without an established pattern that leads to an increase in the size of the susceptible population and that the disappearance of one or both lineages could be related to the genetic variability of the influenza B virus [34,35,36]. In some years, influenza B viruses represent strong bottlenecks in the population and they are generally associated with a high prevalence of influenza A virus [37,38]. Interaction with the influenza A virus might be central in the configuration of the evolutionary dynamics of the influenza B virus and therefore facilitate the change in the dominance or codominance of the lineages between the Victoria and Yamagata lineages [38,39].

During the study period, different patterns of Victoria and Yamagata-lineage B viruses were observed. Regarding the distribution of the different lineages in relation to the age groups of the population studied, our study showed that both the Victoria and Yamagata viruses were more likely to infect patients aged between five and 14 years, which had also been observed in previous studies [32,33,40,41,42]. Moreover, it can be observed that Victoria lineage viruses tend to be distributed according to age with a peak in children under 10 years. Meanwhile, Yamagata lineage viruses frequently show two peaks (in children younger than 10 years and a significant predominance towards individuals over 25 years, preferentially adults over 64 years), as in other countries [12,43,44]. Interestingly, Vijaykrishna and colleagues [12] proposed that age difference between Victoria and Yamagata-lineage infections is thought to be due to differences in the molecular aspects of cellular dynamics, which help the viruses to infect the epithelium of the respiratory tract, while Orsi and colleagues [45] proposed a higher reproductive number (Ro) of the Victoria-lineage viruses, which might reduce the mean age of lineage-specific infections. More studies are required to determine whether there is indeed any difference with either of the B lineages in different age groups. Regarding the distribution of influenza B viruses in relation to the surveillance type, the highest proportion of young children (< 4 years) within SARI patients could be due to different behaviors when a child is found to be sick. Children are more likely to be taken to hospital, especially for SARI cases. It is well known that SARI cases in children could lead to respiratory failure. Respiratory Sincitial Virus (RSV) and human Metapneumovirus (hMPV) significantly increase the risk of SARI, especially in children younger than 24 months. [46]. Similar results have been obtained from other countries [47,48]. The finding from ILI sentinel sites revealed that the influenza positivity rate was high among children aged 5–9 years and became practically null in ages >39 years. Interestingly, and even though ILI surveillance was only done during three of the eight years of the study, the proportion of influenza B found was significantly higher than the observed among SARI cases across different groups of age. Some studies have demonstrated that influenza played an important role in the viral aetiologies of ILI cases [49,50], while other respiratory viruses were essential for the cause of SARI, especially in children [46,51].

Phylogenetic analysis indicated that the HA and NA sequences of influenza B virus could be divided in two distinct lineages: Victoria and Yamagata. The Victoria lineage can be subdivided into V1A and V1B. The V1A clade was the only detected in Uruguay during 2012–2019. The 2012–2017 Uruguayan sequences were genetically related to B/Brisbane/60/2008, the vaccine strain recommended by the WHO to be used in the trivalent vaccine during 2012, 2016, and 2017 for the Southern Hemisphere. In 2019, all of the B/Victoria viruses detected in Uruguay belonged to the V1A clade, encoding a deletion of two amino acids (K162 and N163) in the HA protein.They were grouped within V1A.1 subclade and genetically related to B/Colorado/06/2017, the vaccine strain recommended by the WHO to be used in the trivalent vaccine in 2019 for the Southern Hemisphere. Viruses with the double amino acid deletion in HA were detected in the 2016–2017 Northern Hemisphere season and have continued to circulate since then [52]. Unlike Uruguay, the circulation of these strains has been observed in neighboring countries since 2017, extending even further in 2018 and prevailing in 2019 in the South American region.

The Yamagata lineage can be subdivided into clades 2 and 3. In Uruguay, Yamagata clade 3 was detected since 2014 and the sequences were similar to B/Phuket/3073/2013, the vaccine strain recommended by WHO and included in the trivalent vaccine in 2015 and 2018 for the Southern Hemisphere. Yamagata clade 2 was only found in 2012 in Uruguay, confirming recent reports that indicated the incidence of clade 2 viruses decreased substantially [39]. However, the co-circulation of Yamagata clade 2 and 3 was detected in neighboring countries between 2012–2014. The co-existence of clade 2 and 3 of Yamagata lineage in a region enables these viruses to evolve in multiple dimensions [39]. However, the mixing of viruses from consecutive seasons is less seen in Victoria lineage, rather than in Yamagata lineage viruses [53].

The hemagglutinin represents a primary target for host neutralizing antibodies. More specifically, the HA1 region contains the receptor-binding sites and the most part of antigenic sites, including the 120-loop, the 150-loop, the 160-loop, and the 190-helix and their surrounding regions [21,54]. In this study, several amino acid substitutions were located in the antigenic epitopes of the Uruguayan viruses sequenced, as compared to their specific lineage vaccine strain. In B/Victoria viruses, mutations were mainly observed in the 120-loop. Mutations in the 150-loop were found until 2015 and no mutations occurred in the 190-helix. Variations in the 160-loop were found since 2019 within all of the Uruguayan HA sequenced. Previous studies show that the 120-loop is one of the sites with greater variability and play a crucial role in stabilizing the HA protein structure [21,55]. Insertions or deletions within the 160-loop region might enable influenza B virus to survive longer without undergoing an antigenic shift [56]. Meanwhile, in the B/Yamagata viruses, mutations in the four antigenic sites were only found in 2012 HA sequences. The large number of amino acids substitutions in 2012 could be due to the fact that the HA sequences of that year were of a different clade from the vaccine strain clade that was used in those years. The non-existence of amino acids substitutions at antigenic sites in years after 2012 would be somehow confirming the slowly evolving influenza B/Yamagata viruses.

In some of the years studied, amino acid substitutions were observed outside the known antigenic regions in the HA gene that have only circulated in Uruguay. Moreover, the same samples have unique substitutions in the NA gene. Recent research has suggested the presence of additional antigenic residues outside the known antigenic regions [57]. Amino acid changes in these regions may alter viral antigenicity, and potentially change the evolution direction as new lineages evolve [54]. These mutations may have contributed to increasing viral fitness. If they contributed to antigenic evolution, changes in these positions might have caused conformational changes affecting the antigenic sites. Moreover, it is important that the activity of the two main surface glycoproteins (HA and NA) be balanced to efficiently maintain the capacity for infection and the release of viral particles [58]. Compensatory mutations that maintain the replicative capacities of the virus are necessary [59,60]. It is also imperative that the relative activity of the two proteins is balanced to maintain the ability to infect and release from cells efficiently.

It is noteworthy that 45% of the 2018 Uruguayan B/Yamagata sequences analyzed presented a new potential glycosylation site at position 232 that was also observed in neighboring countries and other regions of the world [61,62].

N-linked glycosylation plays a major role in stabilizing the structure of HA, protecting it from being hydrolyzed by the enzyme and evading antibody recognition. It is known that the diversity in glycosylation in the HA1 epitope produces an antigenic change [21].

Until now, vaccination remains the most effective preventive measure for reducing the incidence and severity of the disease [63]. The selection of the influenza B virus strain for the annual trivalent vaccine is challenging, being low the protective effect of vaccination when the lineage opposite the vaccine or both lineages co-circulate in the population [13,64]. Recent studies in Australia, United States, Europe, Latin America, and the Caribbean show that there is more than one-third of mismatches between the vaccine and the predominant circulating lineage [26,65,66]. Indeed, we show here, that both influenza B lineages circulated almost 2012–2019, in different proportions. Mismatches were found between the vaccines and circulating strains. Our findings evidenced that the level of B vaccine mismatch varied during the eight seasons, with the highest impact being observed in 2015. Our data showed that 28% of the influenza B infections that circulated in Uruguay did not correspond to the lineage of the recommended vaccine strain. The potential impact of vaccine mismatch has been broadly investigated and the findings highlighted the effect on the epidemiology of influenza viruses. Furthermore, it was reported that the vaccine efficacy for influenza B was highly reduced when the circulating lineage was not matched with vaccine lineage [13]. Although Uruguay provided the trivalent vaccine free of charge especially those at high risk, the vaccine coverage remained low (on average 24 % and 30% in subjects < 4 years and >64 years, respectively), as it happened in other countries of the region [28,67]. The lack of availability of vaccination data and antigenic analysis in our study population limit our possibility to draw any inference on this topic. However, the magnitude of the impact of seasonal vaccine mismatch on influenza epidemiology depends on several factors during a given season and the proportion of each influenza B virus lineage circulating, as reported by Reed and colleagues [68]. Quadrivalent vaccines developed, which contain strains of both lineages, can be expected to increase the effectiveness of the vaccine and reduce the morbidity and mortality that are caused by the virus. It seems a logical consequence that children might benefit most from the implementation of specific vaccine-based preventive measures, with potential to reduce the burden of disease in vaccinated and unvaccinated individuals. However so far, only a few countries actually recommend the Quadrivalent influenza vaccination providing the vaccine free of charge [69].

Although information for other internal gene segments may represent an important limitation of this study, the results presented provide evidence of heterogeneity and genetic information for estimating the variability of circulating influenza B viruses in Uruguay and the neighboring countries. Further antigenic analysis is needed for assessing the characteristics of the vaccine candidates and the circulating strains, especially those that possess changes at antigenic and glycosylation sites of the hemagglutinin. Comparing the antigenic relationship and amino acid substitutions help to define the appropriate vaccine strains. Another limitation is that we only examined NA mutations in previously described antigenic sites. Susceptibility testing of patient isolates and antiviral surveillance studies, as well as the functional characterization of molecular markers of drug resistance, are needed. Despite the limitations, the surveillance system for influenza B viruses has been proven useful to describe the epidemiological and evolutionary behavior of influenza B viruses that have circulated in Uruguay during the last years and the results demonstrate that the virus is changing, in line with other observations that were reported from other countries [33].

## 5. Conclusions

This study is one of the first to highlight the prevalence and molecular characteristics of influenza B virus strains, especially the hemagglutinin and neuraminidase genes, which have circulated in Uruguay in recent years. Our findings, like those other studies, confirm the genetic variability of the influenza B viruses and show the importance of systematic epidemiological and molecular surveillance for the effective management of influenza epidemics. Understanding the different evolutionary behavior of the influenza viruses, and how they might interact, is clearly of importance in predicting their future impact on human populations, and may contribute to future vaccine design, and also for better monitoring in surveillance studies.

## Figures and Tables

**Table 1:** Age distribution of influenza B infections according to type of surveillance and influenza B lineages. Numbers of patients are indicated according to age group, discriminating by type of surveillance and influenza B lineages between 2012–2019. Lineage determination in samples *n* = 208. * *p*-value was calculated while using the Pearson chi-square test and the median test.

	Samples Processed	ILI Total	SARI Total	IBV Total	B/Vic	B/Yam	IBV Untyped	* *p*-Value	ILI (+IBV)	SARI (+IBV)	* *p*-Value
Demographic feature											
Mean age in years	20.5	20.5	20.1	22	15.4	29.4			14.3	24.0	
Median age in years	4	10	4	8	6.5	13.5		<0.05	9.5	9	>0.05
Number of patients between											
0–4 years	3210	166	3044	67	39	27	1		9	57	
5–9 years	445	52	393	44	30	14	0		13	31	
10–14 years	145	30	115	26	16	9	1		8	18	
15–19 years	55	10	45	3	2	1	0		2	1	
20–24 years	90	25	65	5	2	2	1		3	2	
25–39 years	284	74	210	18	7	10	1		7	11	
40–49 years	210	49	161	11	2	9	0		1	10	
50–59 years	304	21	283	6	1	5	0		1	5	
60–64 years	177	8	169	3	1	2	0		0	4	
>64 years	834	20	814	34	10	19	5		0	33	
Total	5754	455	5299	217	110	98	9		44	172	

## Supplementary Materials

The following are available online at https://www.mdpi.com/2076-2607/8/4/591/s1.
