# Supplementary material for: Epidemiology and Genetic Variability of Circulating Influenza B Viruses in Uruguay, 2012–2019
Source: Microorganisms. 2020 Apr 19;8(4):591. doi: 10.3390/microorganisms8040591 (PMC7232498; doi:10.3390/microorganisms8040591)
Supplement: Supplementary file 1 [file microorganisms-08-00591-s001.pdf]

Supplementary Materials

We gratefully acknowledge the authors, originating and submitting laboratories of the sequences from GISAID's EpiFlu™ Database on which this research is based. The list is detailed below. All submitters of data may be contacted directly via www.gisaid.org

Segment ID	Segment	Country	Collection date	Isolate name	Originating Lab	Submitting Lab
EPI406238	HA	Argentina	2012-Aug-26	B/Buenos Aires/8833/2012	Instituto Nacional de Enfermedades Infecciosas	National Institute for Medical Research
EPI406325	HA	Argentina	2012-Aug-14	B/Tucuman/A285/2012	Instituto Nacional de Enfermedades Infecciosas	National Institute for Medical Research
EPI543212	HA	Argentina	2014-Jul-02	B/Santa Fe/1079/2014	Instituto Nacional de Enfermedades Infecciosas	National Institute for Medical Research
EPI543214	HA	Argentina	2014-Jul-02	B/Santa Fe/1149/2014	Instituto Nacional de Enfermedades Infecciosas	National Institute for Medical Research
EPI651890	HA	Argentina	2015-Jan-01	B/Argentina/11616/2015	Intituto Nacional de Epidemiologia	Centers for Disease Control and Prevention
EPI807535	HA	Argentina	2016-May-15	B/Argentina/220/2016	Instituto Nacional de Enfermedades Infecciosas	Centers for Disease Control and Prevention
EPI825953	HA	Argentina	2016-Feb-19	B/Argentina/19/2016	Instituto Nacional de Enfermedades Infecciosas	Centers for Disease Control and Prevention
EPI1058963	HA	Argentina	2017-Jul-03	B/Argentina/1744/2017	Instituto Nacional de Enfermedades Infecciosas	Centers for Disease Control and Prevention
EPI1078709	HA	Argentina	2017-Jul-27	B/Argentina/13252/2017	Intituto Nacional de Epidemiologia	Centers for Disease Control and Prevention
EPI1228766	HA	Argentina	2018-Feb-12	B/Argentina/14/2018	Instituto Nacional de Enfermedades Infecciosas	Centers for Disease Control and Prevention
EPI1333555	HA	Argentina	2018-Aug-06	B/Argentina/13785/2018	Intituto Nacional de Epidemiologia	Centers for Disease Control and Prevention
EPI394721	HA	Brazil	2012-Jul-16	B/Natal/119888/2012	National Influenza Center	Centers for Disease Control and Prevention
EPI465903	HA	Brazil	2013-May-06	B/Brazil/374/2013	Instituto Oswaldo Cruz FIOCRUZ	Centers for Disease Control and Prevention
EPI465935	HA	Brazil	2013-May-17	B/Brazil/325/2013	Instituto Oswaldo Cruz FIOCRUZ	Centers for Disease Control and Prevention
EPI543645	HA	Brazil	2014-Jul-10	B/Brazil/1716/2014	Instituto Adolfo Lutz	Centers for Disease Control and Prevention
EPI543660	HA	Brazil	2014-Jul-15	B/Brazil/2282/2014	Instituto Adolfo Lutz	Centers for Disease Control and Prevention
EPI695045	HA	Brazil	2015-Jun-08	B/Brazil/36401/2015	Instituto Adolfo Lutz	Centers for Disease Control and Prevention
EPI714057	HA	Brazil	2015-Sep-15	B/Brazil/0589/2015	Instituto Oswaldo Cruz FIOCRUZ	Centers for Disease Control and Prevention
EPI834113	HA	Brazil	2016-Apr-13	B/Rio Grande Do	National Influenza Center	Centers for Disease Control and Prevention
EPI908342	HA	Brazil	2016-Sep-23	B/Pernambuco/144718-IEC/2016	National Influenza Center	Centers for Disease Control and Prevention
EPI1206891	HA	Brazil	2017-Sep-21	B/Brazil/8134/2017	Instituto Adolfo Lutz	Centers for Disease Control and Prevention
EPI1247581	HA	Brazil	2017-Oct-03	B/Brazil/3889/2017	Instituto Adolfo Lutz	Centers for Disease Control and Prevention
EPI1311172	HA	Brazil	2018-Jul-16	B/Brazil/0379/2018	Instituto Adolfo Lutz	Centers for Disease Control and Prevention
EPI1312924	HA	Brazil	2018-Jul-17	B/Brazil/7103/2018	Instituto Adolfo Lutz	Centers for Disease Control and Prevention
EPI1584217	HA	Brazil	2019-Jan-11	A/Espirito Santo/87/2019	LACEN/ES - Laboratório Central de Saúde Pública do Espírito	Instituto Oswaldo Cruz FIOCRUZ
EPI1574854	HA	Chile	2019-Jul-12	B/Santiago/64801/2019	Instituto de Salud Publica de Chile	Instituto de Salud Publica de Chile
EPI1574886	HA	Chile	2019-Jul-14	B/Santiago/64840/2019	Instituto de Salud Publica de Chile	Instituto de Salud Publica de Chile
EPI368779	HA	Chile	2012-Feb-19	B/Santiago/11896/2012	Instituto de Salud Publica de Chile	Centers for Disease Control and Prevention
EPI378195	HA	Chile	2012-May-29	B/Santiago/38204/2012	Instituto de Salud Publica de Chile	Centers for Disease Control and Prevention
EPI452256	HA	Chile	2013-Apr-09	B/Santiago/23499/2013	Instituto de Salud Publica de Chile	Centers for Disease Control and Prevention
EPI504555	HA	Chile	2013-Dec-06	B/Santiago/80686/2013	Instituto de Salud Publica de Chile	Centers for Disease Control and Prevention
EPI569116	HA	Chile	2014-Nov-01	B/Santiago/71933/2014	Instituto de Salud Publica de Chile	Centers for Disease Control and Prevention
EPI569128	HA	Chile	2014-Nov-08	B/Santiago/73755/2014	Instituto de Salud Publica de Chile	Centers for Disease Control and Prevention
EPI695101	HA	Chile	2015-Oct-22	B/Santiago/75552/2015	Instituto de Salud Publica de Chile	Centers for Disease Control and Prevention
EPI695117	HA	Chile	2015-Oct-17	B/Pucon/75541/2015	Instituto de Salud Publica de Chile	Centers for Disease Control and Prevention
EPI807427	HA	Chile	2016-Mar-16	B/Santiago/20269/2016	Instituto de Salud Publica de Chile	Centers for Disease Control and Prevention
EPI830817	HA	Chile	2016-Jun-13	B/Valparaiso/49570/2016	Instituto de Salud Publica de Chile	Centers for Disease Control and Prevention
EPI1137837	HA	Chile	2017-Oct-02	B/Santiago/75265/2017	Instituto de Salud Publica de Chile	Centers for Disease Control and Prevention
EPI1137973	HA	Chile	2017-Oct-25	B/Santiago/81374/2017	Instituto de Salud Publica de Chile	Centers for Disease Control and Prevention
EPI1261287	HA	Chile	2018-Apr-23	B/Santiago/34517/2018	Instituto de Salud Publica de Chile	Centers for Disease Control and Prevention
EPI1320606	HA	Chile	2018-Jun-27	B/Santiago/53060/2018	Instituto de Salud Publica de Chile	Centers for Disease Control and Prevention
EPI482837	HA	Paraguay	2013-Jun-27	B/Paraguay/022/2013	Central Laboratory of Public Health	Centers for Disease Control and Prevention
EPI544224	HA	Paraguay	2014-Jun-24	B/Paraguay/0068/2014	Central Laboratory of Public Health	Centers for Disease Control and Prevention
EPI562679	HA	Paraguay	2014-Nov-25	B/Paraguay/2429/2014	Central Laboratory of Public Health	Centers for Disease Control and Prevention
EPI708898	HA	Paraguay	2015-Aug-05	B/Paraguay/6712/2015	Central Laboratory of Public Health	Centers for Disease Control and Prevention
EPI753834	HA	Paraguay	2015-Aug-31	B/Paraguay/0905/2015	Central Laboratory of Public Health	Centers for Disease Control and Prevention
EPI830607	HA	Paraguay	2016-Mar-29	B/Paraguay/3296/2016	Central Laboratory of Public Health	Centers for Disease Control and Prevention
EPI892126	HA	Paraguay	2016-Nov-18	B/Paraguay/5243/2016	Central Laboratory of Public Health	Centers for Disease Control and Prevention
EPI1078994	HA	Paraguay	2017-Feb-19	B/Paraguay/3099/2017	Central Laboratory of Public Health	Centers for Disease Control and Prevention
EPI1079018	HA	Paraguay	2017-May-09	B/Paraguay/8072/2017	Central Laboratory of Public Health	Centers for Disease Control and Prevention
EPI1278359	HA	Paraguay	2018-Apr-17	B/Paraguay/0336/2018	Central Laboratory of Public Health	Centers for Disease Control and Prevention
EPI1278415	HA	Paraguay	2018-Jun-18	B/Paraguay/6288/2018	Central Laboratory of Public Health	Centers for Disease Control and Prevention
EPI397176	HA	Uruguay	2012-May-14	B/Uruguay/153/2012	Departamento de Laboratorio de Salud Pública (DLSP)	Centers for Disease Control and Prevention
EPI397496	HA	Uruguay	2012-Jul-19	B/Uruguay/358/2012	Departamento de Laboratorio de Salud Pública (DLSP)	Centers for Disease Control and Prevention
EPI1694428	HA	Uruguay	2012-Aug-18	B/Uruguay/488/2012	Departamento de Laboratorio de Salud Pública (DLSP)	National Influenza Center, Ministry of Health
EPI1694437	HA	Uruguay	2012-Feb-07	B/Uruguay/252/2012	Departamento de Laboratorio de Salud Pública (DLSP)	National Influenza Center, Ministry of Health
EPI1694439	HA	Uruguay	2012-Feb-07	B/Uruguay/266/2012	Departamento de Laboratorio de Salud Pública (DLSP)	National Influenza Center, Ministry of Health
EPI1694441	HA	Uruguay	2012-Apr-07	B/Uruguay/276/2012	Departamento de Laboratorio de Salud Pública (DLSP)	National Influenza Center, Ministry of Health
EPI1694443	HA	Uruguay	2012-Aug-30	B/Uruguay/515/2012	Departamento de Laboratorio de Salud Pública (DLSP)	National Influenza Center, Ministry of Health
EPI543636	HA	Uruguay	2014-Jun-02	B/Uruguay/143/2014	Departamento de Laboratorio de Salud Pública (DLSP)	Centers for Disease Control and Prevention
EPI1694451	HA	Uruguay	2014-Aug-08	B/Uruguay/422/2014	Departamento de Laboratorio de Salud Pública (DLSP)	National Influenza Center, Ministry of Health
EPI1694456	HA	Uruguay	2015-Nov-27	B/Uruguay/372/2015	Departamento de Laboratorio de Salud Pública (DLSP)	National Influenza Center, Ministry of Health
EPI1694458	HA	Uruguay	2015-Oct-23	B/Uruguay/309/2015	Departamento de Laboratorio de Salud Pública (DLSP)	National Influenza Center, Ministry of Health
EPI1034133	HA	Uruguay	2017-May-23	B/Uruguay/106/2017	Departamento de Laboratorio de Salud Pública (DLSP)	Centers for Disease Control and Prevention
EPI1034141	HA	Uruguay	2017-May-25	B/Uruguay/113/2017	Departamento de Laboratorio de Salud Pública (DLSP)	Centers for Disease Control and Prevention
EPI1034148	HA	Uruguay	2017-May-26	B/Uruguay/119/2017	Departamento de Laboratorio de Salud Pública (DLSP)	Centers for Disease Control and Prevention
EPI1034164	HA	Uruguay	2017-May-29	B/Uruguay/126/2017	Departamento de Laboratorio de Salud Pública (DLSP)	Centers for Disease Control and Prevention
EPI1053031	HA	Uruguay	2017-Jun-20	B/Uruguay/403/2017	Departamento de Laboratorio de Salud Pública (DLSP)	Centers for Disease Control and Prevention
EPI1053039	HA	Uruguay	2017-Jun-30	B/Uruguay/527/2017	Departamento de Laboratorio de Salud Pública (DLSP)	Centers for Disease Control and Prevention
EPI1053047	HA	Uruguay	2017-Jun-15	B/Uruguay/338/2017	Departamento de Laboratorio de Salud Pública (DLSP)	Centers for Disease Control and Prevention
EPI1694477	HA	Uruguay	2017-Nov-11	B/Uruguay/1064/2017	Departamento de Laboratorio de Salud Pública (DLSP)	Departamento de Laboratorio de Salud Pública
EPI1311466	HA	Uruguay	2018-Aug-01	B/Uruguay/626/2018	Departamento de Laboratorio de Salud Pública (DLSP)	National Influenza Center, Ministry of Health
EPI1311467	HA	Uruguay	2018-Aug-23	B/Uruguay/835/2018	Departamento de Laboratorio de Salud Pública (DLSP)	National Influenza Center, Ministry of Health
EPI1311468	HA	Uruguay	2018-Aug-29	B/Uruguay/899/2018	Departamento de Laboratorio de Salud Pública (DLSP)	National Influenza Center, Ministry of Health
EPI1312796	HA	Uruguay	2018-Jul-24	B/Uruguay/571/2018	Departamento de Laboratorio de Salud Pública (DLSP)	Centers for Disease Control and Prevention
EPI1312804	HA	Uruguay	2018-Jul-19	B/Uruguay/553/2018	Departamento de Laboratorio de Salud Pública (DLSP)	Centers for Disease Control and Prevention
EPI1312812	HA	Uruguay	2018-Feb-20	B/Uruguay/033/2018	Departamento de Laboratorio de Salud Pública (DLSP)	Centers for Disease Control and Prevention
EPI1694495	HA	Uruguay	2018-Sep-18	B/Uruguay/1054/2018	Departamento de Laboratorio de Salud Pública (DLSP)	National Influenza Center, Ministry of Health
EPI1694497	HA	Uruguay	2018-Sep-12	B/Uruguay/1005/2018	Departamento de Laboratorio de Salud Pública (DLSP)	National Influenza Center, Ministry of Health
EPI1694499	HA	Uruguay	2018-Aug-22	B/Uruguay/816/2018	Departamento de Laboratorio de Salud Pública (DLSP)	National Influenza Center, Ministry of Health
EPI1694501	HA	Uruguay	2018-Aug-06	B/Uruguay/727/2018	Departamento de Laboratorio de Salud Pública (DLSP)	National Influenza Center, Ministry of Health
EPI1694503	HA	Uruguay	2018-Sep-21	B/Uruguay/1070/2018	Departamento de Laboratorio de Salud Pública (DLSP)	National Influenza Center, Ministry of Health
EPI406250	HA	Argentina	2012-Aug-06	B/Formosa/V2367/2012	Instituto Nacional de Enfermedades Infecciosas	National Institute for Medical Research
EPI406282	HA	Argentina	2012-Jul-06	B/Buenos Aires/770/2012	Instituto Nacional de Enfermedades Infecciosas	National Institute for Medical Research
EPI540572	HA	Argentina	2014-Jul-02	B/Argentina/154/2014	Instituto Nacional de Enfermedades Infecciosas	Centers for Disease Control and Prevention
EPI543161	HA	Argentina	2014-Jun-03	B/Buenos Aires/10986152/2014	Instituto Nacional de Enfermedades Infecciosas	National Institute for Medical Research
EPI651978	HA	Argentina	2015-Jul-18	B/Argentina/11461/2015	Intituto Nacional de Epidemiologia	Centers for Disease Control and Prevention
EPI831248	HA	Argentina	2016-May-20	B/Argentina/268/2016	Instituto Nacional de Enfermedades Infecciosas	Centers for Disease Control and Prevention
EPI1058995	HA	Argentina	2017-Jul-06	B/Argentina/2110/2017	Instituto Nacional de Enfermedades Infecciosas	Centers for Disease Control and Prevention
EPI1078716	HA	Argentina	2017-Jul-27	B/Argentina/13339/2017	Intituto Nacional de Epidemiologia	Centers for Disease Control and Prevention
EPI1247025	HA	Argentina	2018-Jan-29	B/Argentina/9/2018	Instituto Nacional de Enfermedades Infecciosas	Centers for Disease Control and Prevention
EPI1311235	HA	Argentina	2018-Jul-06	B/Argentina/13738/2018	Intituto Nacional de Epidemiologia	Centers for Disease Control and Prevention
EPI1529644	HA	Brazil	2019-Jul-07	B/Sao Paulo/1211465-IAL/2019	Instituto Adolfo Lutz	Instituto Adolfo Lutz
EPI1602473	HA	Brazil	2019-Jun-10	B/Sao Paulo/1187418-IAL/2019	Instituto Adolfo Lutz	Centers for Disease Control and Prevention
EPI387720	HA	Brazil	2012-Feb-16	B/Brazil/0944/2012	Instituto Adolfo Lutz	Centers for Disease Control and Prevention
EPI468090	HA	Brazil	2013-Jun-25	B/Brazil/6004/2013	Instituto Adolfo Lutz	Centers for Disease Control and Prevention
EPI473777	HA	Brazil	2013-Aug-14	B/Brazil/3742/2013	Instituto Adolfo Lutz	Centers for Disease Control and Prevention
EPI536552	HA	Brazil	2014-Feb-27	B/Brazil/2920/2014	Instituto Adolfo Lutz	Centers for Disease Control and Prevention
EPI536584	HA	Brazil	2014-Mar-25	B/Brazil/5137/2014	Instituto Adolfo Lutz	Centers for Disease Control and Prevention
EPI642294	HA	Brazil	2015-Apr-14	B/Brazil/6297/2015	Instituto Adolfo Lutz	Centers for Disease Control and Prevention
EPI695357	HA	Brazil	2015-Aug-09	B/Brazil/80287/2015	Instituto Adolfo Lutz	Centers for Disease Control and Prevention
EPI831041	HA	Brazil	2016-May-11	B/Brazil/0496/2016	Instituto Oswaldo Cruz FIOCRUZ	Centers for Disease Control and Prevention
EPI912634	HA	Brazil	2016-Dec-13	B/Ceara/144952-IEC/2016	National Influenza Center	Centers for Disease Control and Prevention
EPI1078748	HA	Brazil	2017-May-26	B/Brazil/6128/2017	Instituto Adolfo Lutz	Centers for Disease Control and Prevention
EPI1089703	HA	Brazil	2017-May-25	B/Brazil/4558/2017	Instituto Adolfo Lutz	Centers for Disease Control and Prevention
EPI1311088	HA	Brazil	2018-Jul-25	B/Brazil/2470/2018	Instituto Adolfo Lutz	Centers for Disease Control and Prevention
EPI1312993	HA	Brazil	2018-Aug-10	B/Brazil/4835/2018	Instituto Adolfo Lutz	Centers for Disease Control and Prevention
EPI1610199	HA	Brazil	2019-Jul-08	B/Bahia/735/2019	Instituto Oswaldo Cruz FIOCRUZ	Centers for Disease Control and Prevention
EPI1584235	HA	Brazil	2019-Apr-04	B/Bahia/148/2019	Laboratório Central de Saúde Pública Professor Gonçalo Moniz	Instituto Oswaldo Cruz FIOCRUZ
EPI555041	HA	Chile	2013-Dec-30	B/Concepcion/264/2014	Instituto de Salud Publica de Chile	Instituto de Salud Publica de Chile
EPI378192	HA	Chile	2012-May-14	B/Santiago/34960/2012	Instituto de Salud Publica de Chile	Centers for Disease Control and Prevention
EPI395213	HA	Chile	2012-Jul-10	B/Santiago/49303/2012	Instituto de Salud Publica de Chile	Centers for Disease Control and Prevention
EPI508380	HA	Chile	2013-Dec-10	B/Santiago/82832/2013	Instituto de Salud Publica de Chile	Centers for Disease Control and Prevention
EPI508402	HA	Chile	2013-Dec-11	B/Puerto Montt/81199/2013	Instituto de Salud Publica de Chile	Centers for Disease Control and Prevention
EPI565203	HA	Chile	2014-Oct-25	B/Santiago/69223/2014	Instituto de Salud Publica de Chile	Centers for Disease Control and Prevention
EPI695125	HA	Chile	2015-Nov-11	B/Valparaiso/81181/2015	Instituto de Salud Publica de Chile	Centers for Disease Control and Prevention
EPI695213	HA	Chile	2015-Nov-10	B/Santiago/80460/2015	Instituto de Salud Publica de Chile	Centers for Disease Control and Prevention
EPI830769	HA	Chile	2016-Jun-24	B/Santiago/44313/2016	Instituto de Salud Publica de Chile	Centers for Disease Control and Prevention
EPI830793	HA	Chile	2016-Jul-04	B/Santiago/45998/2016	Instituto de Salud Publica de Chile	Centers for Disease Control and Prevention
EPI1008168	HA	Chile	2017-Mar-26	B/Santiago/25385/2017	Instituto de Salud Publica de Chile	Centers for Disease Control and Prevention
EPI1052824	HA	Chile	2017-Jul-10	B/Santiago/54067/2017	Instituto de Salud Publica de Chile	Centers for Disease Control and Prevention
EPI1253949	HA	Chile	2018-Apr-24	B/Santiago/34511/2018	Instituto de Salud Publica de Chile	Centers for Disease Control and Prevention

Segment ID	Segment	Country	Collection date	Isolate name	Originating Lab	Submitting Lab
EPI1273538	HA	Chile	2018-Jun-22	B/Santiago/51375/2018	Instituto de Salud Publica de Chile	Centers for Disease Control and Prevention
EPI1585988	HA	Chile	2019-Jul-09	B/Santiago/62161/2019	Instituto de Salud Publica de Chile	Centers for Disease Control and Prevention
EPI1585983	HA	Chile	2019-Jul-04	B/Talca/63221/2019	Instituto de Salud Publica de Chile	Centers for Disease Control and Prevention
EPI387742	HA	Paraguay	2012-Jan-01	B/Paraguay/190/2012	Central Laboratory of Public Health	Centers for Disease Control and Prevention
EPI391319	HA	Paraguay	2012-Jun-04	B/Paraguay/252/2012	Central Laboratory of Public Health	Centers for Disease Control and Prevention
EPI565078	HA	Paraguay	2014-Nov-13	B/Paraguay/672/2014	Central Laboratory of Public Health	Centers for Disease Control and Prevention
EPI565181	HA	Paraguay	2014-Dec-09	B/Paraguay/4674/2014	Central Laboratory of Public Health	Centers for Disease Control and Prevention
EPI642966	HA	Paraguay	2015-May-11	B/Paraguay/1131/2015	Central Laboratory of Public Health	Centers for Disease Control and Prevention
EPI642982	HA	Paraguay	2015-Jun-03	B/Paraguay/5133/2015	Central Laboratory of Public Health	Centers for Disease Control and Prevention
EPI892102	HA	Paraguay	2016-Sep-20	B/Paraguay/2377/2016	Central Laboratory of Public Health	Centers for Disease Control and Prevention
EPI892118	HA	Paraguay	2016-Oct-08	B/Paraguay/5894/2016	Central Laboratory of Public Health	Centers for Disease Control and Prevention
EPI1079010	HA	Paraguay	2017-Jan-20	B/Paraguay/8037/2017	Central Laboratory of Public Health	Centers for Disease Control and Prevention
EPI1278351	HA	Paraguay	2018-May-23	B/Paraguay/0620/2018	Central Laboratory of Public Health	Centers for Disease Control and Prevention
EPI1278407	HA	Paraguay	2018-May-09	B/Paraguay/7301/2018	Central Laboratory of Public Health	Centers for Disease Control and Prevention
EPI1694435	HA	Uruguay	2012-Jun-16	B/Uruguay/236/2012	Departamento de Laboratorio de Salud Pública (DLSP)	National Influenza Center, Ministry of Health
EPI1694445	HA	Uruguay	2012-Nov-07	B/Uruguay/311/2012	Departamento de Laboratorio de Salud Pública (DLSP)	National Influenza Center, Ministry of Health
EPI1694132	HA	Uruguay	2012-Jun-30	B/Uruguay/257/2012	Departamento de Laboratorio de Salud Pública (DLSP)	National Influenza Center, Ministry of Health
EPI1694430	HA	Uruguay	2012-Nov-07	B/Uruguay/312/2012	Departamento de Laboratorio de Salud Pública (DLSP)	National Influenza Center, Ministry of Health
EPI468103	NA	Uruguay	2013-Jun-10	B/Uruguay/177/2013	Departamento de Laboratorio de Salud Pública (DLSP)	Centers for Disease Control and Prevention
EPI1694446	HA	Uruguay	2013-Dec-18	B/Uruguay/864/2013	Departamento de Laboratorio de Salud Pública (DLSP)	National Influenza Center, Ministry of Health
EPI1694447	HA	Uruguay	2014-Aug-15	B/Uruguay/406/2014	Departamento de Laboratorio de Salud Pública (DLSP)	National Influenza Center, Ministry of Health
EPI1694449	HA	Uruguay	2014-Dec-09	B/Uruguay/578/2014	Departamento de Laboratorio de Salud Pública (DLSP)	National Influenza Center, Ministry of Health
EPI662228	NA	Uruguay	2015-Aug-15	B/Uruguay/98/2015	Departamento de Laboratorio de Salud Pública (DLSP)	Centers for Disease Control and Prevention
EPI662235	NA	Uruguay	2015-Aug-17	B/Uruguay/102/2015	Departamento de Laboratorio de Salud Pública (DLSP)	Centers for Disease Control and Prevention
EPI1694463	HA	Uruguay	2015-Nov-17	B/Uruguay/345/2015	Departamento de Laboratorio de Salud Pública (DLSP)	National Influenza Center, Ministry of Health
EPI1694465	HA	Uruguay	2015-Jun-11	B/Uruguay/335/2015	Departamento de Laboratorio de Salud Pública (DLSP)	National Influenza Center, Ministry of Health
EPI1694453	HA	Uruguay	2015-Aug-17	B/Uruguay/105/2015	Departamento de Laboratorio de Salud Pública (DLSP)	National Influenza Center, Ministry of Health
EPI1694460	HA	Uruguay	2015-Oct-19	B/Uruguay/289/2015	Departamento de Laboratorio de Salud Pública (DLSP)	National Influenza Center, Ministry of Health
EPI662229	HA	Uruguay	2015-Aug-15	B/Uruguay/98/2015	Departamento de Laboratorio de Salud Pública (DLSP)	Centers for Disease Control and Prevention
EPI1694467	HA	Uruguay	2015-Mar-12	B/Uruguay/384/2015	Departamento de Laboratorio de Salud Pública (DLSP)	National Influenza Center, Ministry of Health
EPI1694454	HA	Uruguay	2015-Aug-13	B/Uruguay/94/2015	Departamento de Laboratorio de Salud Pública (DLSP)	National Influenza Center, Ministry of Health
EPI662236	HA	Uruguay	2015-Aug-17	B/Uruguay/102/2015	Departamento de Laboratorio de Salud Pública (DLSP)	Centers for Disease Control and Prevention
EPI826797	HA	Uruguay	2016-Jan-08	B/Uruguay/02/2016	Departamento de Laboratorio de Salud Pública (DLSP)	Centers for Disease Control and Prevention
EPI1694468	HA	Uruguay	2016-Aug-08	B/Uruguay/934/2016	Departamento de Laboratorio de Salud Pública (DLSP)	National Influenza Center, Ministry of Health
EPI1694469	HA	Uruguay	2016-Aug-09	B/Uruguay/789/2016	Departamento de Laboratorio de Salud Pública (DLSP)	National Influenza Center, Ministry of Health
EPI1034156	HA	Uruguay	2017-May-30	B/Uruguay/124/2017	Departamento de Laboratorio de Salud Pública (DLSP)	Centers for Disease Control and Prevention
EPI1034172	HA	Uruguay	2017-May-31	B/Uruguay/137/2017	Departamento de Laboratorio de Salud Pública (DLSP)	Centers for Disease Control and Prevention
EPI1034180	HA	Uruguay	2017-May-31	B/Uruguay/138/2017	Departamento de Laboratorio de Salud Pública (DLSP)	Centers for Disease Control and Prevention
EPI1034188	HA	Uruguay	2017-May-31	B/Uruguay/140/2017	Departamento de Laboratorio de Salud Pública (DLSP)	Centers for Disease Control and Prevention
EPI1053015	HA	Uruguay	2017-Jun-30	B/Uruguay/511/2017	Departamento de Laboratorio de Salud Pública (DLSP)	Centers for Disease Control and Prevention
EPI1053023	HA	Uruguay	2017-Jun-19	B/Uruguay/379/2017	Departamento de Laboratorio de Salud Pública (DLSP)	Centers for Disease Control and Prevention
EPI1053055	HA	Uruguay	2017-Jun-20	B/Uruguay/383/2017	Departamento de Laboratorio de Salud Pública (DLSP)	Centers for Disease Control and Prevention
EPI1694471	HA	Uruguay	2017-Aug-21	B/Uruguay/830/2017	Departamento de Laboratorio de Salud Pública (DLSP)	National Influenza Center, Ministry of Health
EPI1694473	HA	Uruguay	2017-Jan-08	B/Uruguay/700/2017	Departamento de Laboratorio de Salud Pública (DLSP)	National Influenza Center, Ministry of Health
EPI1694475	HA	Uruguay	2017-Jul-24	B/Uruguay/659/2017	Departamento de Laboratorio de Salud Pública (DLSP)	National Influenza Center, Ministry of Health
EPI1694770	HA	Uruguay	2019-Sep-17	B/Uruguay/708/2019	Departamento de Laboratorio de Salud Pública (DLSP)	National Influenza Center, Ministry of Health
EPI1694769	HA	Uruguay	2019-Oct-16	B/Uruguay/748/2019	Departamento de Laboratorio de Salud Pública (DLSP)	National Influenza Center, Ministry of Health
EPI1694768	HA	Uruguay	2019-Oct-21	B/Uruguay/753/2019	Departamento de Laboratorio de Salud Pública (DLSP)	National Influenza Center, Ministry of Health
EPI406251	NA	Argentina	2012-Aug-06	B/Formosa/V2367/2012	Instituto Nacional de Enfermedades Infecciosas	National Institute for Medical Research
EPI904580	NA	Argentina	2012-Jul-06	B/Buenos Aires/770/2012	Instituto Nacional de Enfermedades Infecciosas	Crick Worldwide Influenza Centre
EPI540571	NA	Argentina	2014-Jul-02	B/Argentina/154/2014	Instituto Nacional de Enfermedades Infecciosas	Centers for Disease Control and Prevention
EPI543162	NA	Argentina	2014-Jun-03	B/Buenos Aires/10986152/2014	Instituto Nacional de Enfermedades Infecciosas	National Institute for Medical Research
EPI651977	NA	Argentina	2015-Jul-18	B/Argentina/11461/2015	Intituto Nacional de Epidemiologia	Centers for Disease Control and Prevention
EPI831247	NA	Argentina	2016-May-20	B/Argentina/268/2016	Instituto Nacional de Enfermedades Infecciosas	Centers for Disease Control and Prevention
EPI1058994	NA	Argentina	2017-Jul-06	B/Argentina/2110/2017	Instituto Nacional de Enfermedades Infecciosas	Centers for Disease Control and Prevention
EPI1078715	NA	Argentina	2017-Jul-27	B/Argentina/13339/2017	Intituto Nacional de Epidemiologia	Centers for Disease Control and Prevention
EPI1247024	NA	Argentina	2018-Jan-29	B/Argentina/9/2018	Instituto Nacional de Enfermedades Infecciosas	Centers for Disease Control and Prevention
EPI1311234	NA	Argentina	2018-Jul-06	B/Argentina/13738/2018	Intituto Nacional de Epidemiologia	Centers for Disease Control and Prevention
EPI387719	NA	Brazil	2012-Feb-16	B/Brazil/0944/2012	Instituto Adolfo Lutz	Centers for Disease Control and Prevention
EPI468089	NA	Brazil	2013-Jun-25	B/Brazil/6004/2013	Instituto Adolfo Lutz	Centers for Disease Control and Prevention
EPI473776	NA	Brazil	2013-Aug-14	B/Brazil/3742/2013	Instituto Adolfo Lutz	Centers for Disease Control and Prevention
EPI536551	NA	Brazil	2014-Feb-27	B/Brazil/2920/2014	Instituto Adolfo Lutz	Centers for Disease Control and Prevention
EPI536583	NA	Brazil	2014-Mar-25	B/Brazil/5137/2014	Instituto Adolfo Lutz	Centers for Disease Control and Prevention
EPI642293	NA	Brazil	2015-Apr-14	B/Brazil/6297/2015	Instituto Adolfo Lutz	Centers for Disease Control and Prevention
EPI695356	NA	Brazil	2015-Aug-09	B/Brazil/80287/2015	Instituto Adolfo Lutz	Centers for Disease Control and Prevention
EPI831040	NA	Brazil	2016-May-11	B/Brazil/0496/2016	Instituto Oswaldo Cruz FIOCRUZ	Centers for Disease Control and Prevention
EPI912633	NA	Brazil	2016-Dec-13	B/Ceara/144952-IEC/2016	National Influenza Center	Centers for Disease Control and Prevention
EPI1078747	NA	Brazil	2017-May-26	B/Brazil/6128/2017	Instituto Adolfo Lutz	Centers for Disease Control and Prevention
EPI1089702	NA	Brazil	2017-May-25	B/Brazil/4558/2017	Instituto Adolfo Lutz	Centers for Disease Control and Prevention
EPI1311087	NA	Brazil	2018-Jul-25	B/Brazil/2470/2018	Instituto Adolfo Lutz	Centers for Disease Control and Prevention
EPI1312992	NA	Brazil	2018-Aug-10	B/Brazil/4835/2018	Instituto Adolfo Lutz	Centers for Disease Control and Prevention
EPI1602480	NA	Brazil	2019-Jun-12	B/Sao Paulo/1190756-IAL/2019	Instituto Adolfo Lutz	Centers for Disease Control and Prevention
EPI1602496	NA	Brazil	2019-Jul-01	B/Sao Paulo/1211465-IAL/2019	Instituto Adolfo Lutz	Centers for Disease Control and Prevention
EPI1602472	NA	Brazil	2019-Jun-10	B/Sao Paulo/1187418-IAL/2019	Instituto Adolfo Lutz	Centers for Disease Control and Prevention
EPI1610198	NA	Brazil	2019-Jul-08	B/Bahia/735/2019	Instituto Oswaldo Cruz FIOCRUZ	Centers for Disease Control and Prevention
EPI566830	NA	Chile	2013-Dec-30	B/Concepcion/264/2014	Instituto de Salud Publica de Chile	Instituto de Salud Publica de Chile
EPI378191	NA	Chile	2012-May-14	B/Santiago/34960/2012	Instituto de Salud Publica de Chile	Centers for Disease Control and Prevention
EPI395212	NA	Chile	2012-Jul-10	B/Santiago/49303/2012	Instituto de Salud Publica de Chile	Centers for Disease Control and Prevention
EPI508379	NA	Chile	2013-Dec-10	B/Santiago/82832/2013	Instituto de Salud Publica de Chile	Centers for Disease Control and Prevention
EPI508401	NA	Chile	2013-Dec-11	B/Puerto Montt/81199/2013	Instituto de Salud Publica de Chile	Centers for Disease Control and Prevention
EPI695124	NA	Chile	2015-Nov-11	B/Valparaiso/81181/2015	Instituto de Salud Publica de Chile	Centers for Disease Control and Prevention
EPI695212	NA	Chile	2015-Nov-10	B/Santiago/80460/2015	Instituto de Salud Publica de Chile	Centers for Disease Control and Prevention
EPI830768	NA	Chile	2016-Jun-24	B/Santiago/44313/2016	Instituto de Salud Publica de Chile	Centers for Disease Control and Prevention
EPI830792	NA	Chile	2016-Jul-04	B/Santiago/45998/2016	Instituto de Salud Publica de Chile	Centers for Disease Control and Prevention
EPI1008167	NA	Chile	2017-Mar-26	B/Santiago/25385/2017	Instituto de Salud Publica de Chile	Centers for Disease Control and Prevention
EPI1052823	NA	Chile	2017-Jul-10	B/Santiago/54067/2017	Instituto de Salud Publica de Chile	Centers for Disease Control and Prevention
EPI1253948	NA	Chile	2018-Apr-24	B/Santiago/34511/2018	Instituto de Salud Publica de Chile	Centers for Disease Control and Prevention
EPI1273537	NA	Chile	2018-Jun-22	B/Santiago/51375/2018	Instituto de Salud Publica de Chile	Centers for Disease Control and Prevention
EPI1585987	NA	Chile	2019-Jul-09	B/Santiago/62161/2019	Instituto de Salud Publica de Chile	Centers for Disease Control and Prevention
EPI1584242	NA	Brazil	2019-Apr-13	B/Parana/206/2019	Laboratório Central do Estado do Paraná - LACEN/PR	Instituto Oswaldo Cruz FIOCRUZ
EPI1584222	NA	Brazil	2019-Mar-12	B/Parana/115/2019	Laboratório Central do Estado do Paraná - LACEN/PR	Instituto Oswaldo Cruz FIOCRUZ
EPI1585982	NA	Chile	2019-Jul-04	B/Talca/63221/2019	Instituto de Salud Publica de Chile	Centers for Disease Control and Prevention
EPI387741	NA	Paraguay	2012-Jan-01	B/Paraguay/190/2012	Central Laboratory of Public Health	Centers for Disease Control and Prevention
EPI392884	NA	Paraguay	2012-Jun-04	B/Paraguay/252/2012	Central Laboratory of Public Health	Centers for Disease Control and Prevention
EPI565077	NA	Paraguay	2014-Nov-13	B/Paraguay/672/2014	Central Laboratory of Public Health	Centers for Disease Control and Prevention
EPI565180	NA	Paraguay	2014-Dec-09	B/Paraguay/4674/2014	Central Laboratory of Public Health	Centers for Disease Control and Prevention
EPI642965	NA	Paraguay	2015-May-11	B/Paraguay/1131/2015	Central Laboratory of Public Health	Centers for Disease Control and Prevention
EPI642981	NA	Paraguay	2015-Jun-03	B/Paraguay/5133/2015	Central Laboratory of Public Health	Centers for Disease Control and Prevention
EPI892101	NA	Paraguay	2016-Sep-20	B/Paraguay/2377/2016	Central Laboratory of Public Health	Centers for Disease Control and Prevention
EPI892117	NA	Paraguay	2016-Oct-08	B/Paraguay/5894/2016	Central Laboratory of Public Health	Centers for Disease Control and Prevention
EPI1079009	NA	Paraguay	2017-Jan-20	B/Paraguay/8037/2017	Central Laboratory of Public Health	Centers for Disease Control and Prevention
EPI1278350	NA	Paraguay	2018-May-23	B/Paraguay/0620/2018	Central Laboratory of Public Health	Centers for Disease Control and Prevention
EPI1278406	NA	Paraguay	2018-May-09	B/Paraguay/7301/2018	Central Laboratory of Public Health	Centers for Disease Control and Prevention
EPI468103	NA	Uruguay	2013-Jun-10	B/Uruguay/177/2013	Departamento de Laboratorio de Salud Pública (DLSP)	Centers for Disease Control and Prevention
EPI662228	NA	Uruguay	2015-Aug-15	B/Uruguay/98/2015	Departamento de Laboratorio de Salud Pública (DLSP)	Centers for Disease Control and Prevention
EPI662235	NA	Uruguay	2015-Aug-17	B/Uruguay/102/2015	Departamento de Laboratorio de Salud Pública (DLSP)	Centers for Disease Control and Prevention
EPI1694436	NA	Uruguay	2012-Jun-16	B/Uruguay/236/2012	Departamento de Laboratorio de Salud Pública (DLSP)	National Influenza Center, Ministry of Health
EPI1694448	NA	Uruguay	2014-Aug-15	B/Uruguay/406/2014	Departamento de Laboratorio de Salud Pública (DLSP)	National Influenza Center, Ministry of Health
EPI1694450	NA	Uruguay	2014-Dec-09	B/Uruguay/578/2014	Departamento de Laboratorio de Salud Pública (DLSP)	National Influenza Center, Ministry of Health
EPI1694461	NA	Uruguay	2015-Oct-19	B/Uruguay/289/2015	Departamento de Laboratorio de Salud Pública (DLSP)	National Influenza Center, Ministry of Health
EPI1694462	NA	Uruguay	2015-Nov-17	B/Uruguay/342/2015	Departamento de Laboratorio de Salud Pública (DLSP)	National Influenza Center, Ministry of Health
EPI1694464	NA	Uruguay	2015-Nov-17	B/Uruguay/345/2015	Departamento de Laboratorio de Salud Pública (DLSP)	National Influenza Center, Ministry of Health
EPI1694466	NA	Uruguay	2015-Jun-11	B/Uruguay/335/2015	Departamento de Laboratorio de Salud Pública (DLSP)	National Influenza Center, Ministry of Health
EPI1694470	NA	Uruguay	2016-Aug-09	B/Uruguay/789/2016	Departamento de Laboratorio de Salud Pública (DLSP)	National Influenza Center, Ministry of Health
EPI826796	NA	Uruguay	2016-Jan-08	B/Uruguay/02/2016	Departamento de Laboratorio de Salud Pública (DLSP)	Centers for Disease Control and Prevention
EPI1034155	NA	Uruguay	2017-May-30	B/Uruguay/124/2017	Departamento de Laboratorio de Salud Pública (DLSP)	Centers for Disease Control and Prevention
EPI1034171	NA	Uruguay	2017-May-31	B/Uruguay/137/2017	Departamento de Laboratorio de Salud Pública (DLSP)	Centers for Disease Control and Prevention
EPI1034179	NA	Uruguay	2017-May-31	B/Uruguay/138/2017	Departamento de Laboratorio de Salud Pública (DLSP)	Centers for Disease Control and Prevention
EPI1034187	NA	Uruguay	2017-May-31	B/Uruguay/140/2017	Departamento de Laboratorio de Salud Pública (DLSP)	Centers for Disease Control and Prevention
EPI1053014	NA	Uruguay	2017-Jun-30	B/Uruguay/511/2017	Departamento de Laboratorio de Salud Pública (DLSP)	Centers for Disease Control and Prevention
EPI1053022	NA	Uruguay	2017-Jun-19	B/Uruguay/379/2017	Departamento de Laboratorio de Salud Pública (DLSP)	Centers for Disease Control and Prevention
EPI1053054	NA	Uruguay	2017-Jun-20	B/Uruguay/383/2017	Departamento de Laboratorio de Salud Pública (DLSP)	Centers for Disease Control and Prevention
EPI1694472	NA	Uruguay	2017-Aug-21	B/Uruguay/830/2017	Departamento de Laboratorio de Salud Pública (DLSP)	National Influenza Center, Ministry of Health
EPI1694474	NA	Uruguay	2017-Jan-08	B/Uruguay/700/2017	Departamento de Laboratorio de Salud Pública (DLSP)	National Influenza Center, Ministry of Health
EPI1694493	NA	Uruguay	2017-Jul-28	B/Uruguay/673/2017	Departamento de Laboratorio de Salud Pública (DLSP)	National Influenza Center, Ministry of Health
EPI1694764	NA	Uruguay	2019-Sep-17	B/Uruguay/708/2019	Departamento de Laboratorio de Salud Pública (DLSP)	National Influenza Center, Ministry of Health
EPI1694762	NA	Uruguay	2019-Oct-16	B/Uruguay/748/2019	Departamento de Laboratorio de Salud Pública (DLSP)	National Influenza Center, Ministry of Health
EPI1694763	NA	Uruguay	2019-Oct-21	B/Uruguay/753/2019	Departamento de Laboratorio de Salud Pública (DLSP)	National Influenza Center, Ministry of Health
EPI406239	NA	Argentina	2012-Aug-26	B/Buenos Aires/8833/2012	Instituto Nacional de Enfermedades Infecciosas	National Institute for Medical Research
EPI406326	NA	Argentina	2012-Aug-14	B/Tucuman/A285/2012	Instituto Nacional de Enfermedades Infecciosas	National Institute for Medical Research
EPI543213	NA	Argentina	2014-Jul-02	B/Santa Fe/1079/2014	Instituto Nacional de Enfermedades Infecciosas	National Institute for Medical Research
EPI543215	NA	Argentina	2014-Jul-02	B/Santa Fe/1149/2014	Instituto Nacional de Enfermedades Infecciosas	National Institute for Medical Research
EPI651889	NA	Argentina	2015-Jan-01	B/Argentina/11616/2015	Intituto Nacional de Epidemiologia	Centers for Disease Control and Prevention
EPI807534	NA	Argentina	2016-May-15	B/Argentina/220/2016	Instituto Nacional de Enfermedades Infecciosas	Centers for Disease Control and Prevention
EPI825952	NA	Argentina	2016-Feb-19	B/Argentina/19/2016	Instituto Nacional de Enfermedades Infecciosas	Centers for Disease Control and Prevention
EPI1058962	NA	Argentina	2017-Jul-03	B/Argentina/1744/2017	Instituto Nacional de Enfermedades Infecciosas	Centers for Disease Control and Prevention
EPI1078708	NA	Argentina	2017-Jul-27	B/Argentina/13252/2017	Intituto Nacional de Epidemiologia	Centers for Disease Control and Prevention
EPI1228765	NA	Argentina	2018-Feb-12	B/Argentina/14/2018	Instituto Nacional de Enfermedades Infecciosas	Centers for Disease Control and Prevention

Segment ID	Segment	Country	Collection date	Isolate name	Originating Lab	Submitting Lab
EPI1333554	NA	Argentina	2018-Aug-06	B/Argentina/13785/2018	Intituto Nacional de Epidemiologia	Centers for Disease Control and Prevention
EPI394720	NA	Brazil	2012-Jul-16	B/Natal/119888/2012	National Influenza Center	Centers for Disease Control and Prevention
EPI465902	NA	Brazil	2013-May-06	B/Brazil/374/2013	Instituto Oswaldo Cruz FIOCRUZ	Centers for Disease Control and Prevention
EPI465934	NA	Brazil	2013-May-17	B/Brazil/325/2013	Instituto Oswaldo Cruz FIOCRUZ	Centers for Disease Control and Prevention
EPI543644	NA	Brazil	2014-Jul-10	B/Brazil/1716/2014	Instituto Adolfo Lutz	Centers for Disease Control and Prevention
EPI543659	NA	Brazil	2014-Jul-15	B/Brazil/2282/2014	Instituto Adolfo Lutz	Centers for Disease Control and Prevention
EPI695044	NA	Brazil	2015-Jun-08	B/Brazil/36401/2015	Instituto Adolfo Lutz	Centers for Disease Control and Prevention
EPI714056	NA	Brazil	2015-Sep-15	B/Brazil/0589/2015	Instituto Oswaldo Cruz FIOCRUZ	Centers for Disease Control and Prevention
EPI834112	NA	Brazil	2016-Apr-13	B/Rio Grande Do	National Influenza Center	Centers for Disease Control and Prevention
EPI908341	NA	Brazil	2016-Sep-23	B/Pernambuco/144718-IEC/2016	National Influenza Center	Centers for Disease Control and Prevention
EPI1206890	NA	Brazil	2017-Sep-21	B/Brazil/8134/2017	Instituto Adolfo Lutz	Centers for Disease Control and Prevention
EPI1247580	NA	Brazil	2017-Oct-03	B/Brazil/3889/2017	Instituto Adolfo Lutz	Centers for Disease Control and Prevention
EPI1311171	NA	Brazil	2018-Jul-16	B/Brazil/0379/2018	Instituto Adolfo Lutz	Centers for Disease Control and Prevention
EPI1312923	NA	Brazil	2018-Jul-17	B/Brazil/7103/2018	Instituto Adolfo Lutz	Centers for Disease Control and Prevention
EPI1584218	NA	Brazil	2019-Jan-11	A/Espirito Santo/87/2019	LACEN/ES - Laboratório Central de Saúde Pública do Espírito	Instituto Oswaldo Cruz FIOCRUZ
EPI1574853	NA	Chile	2019-Jul-12	B/Santiago/64801/2019	Instituto de Salud Publica de Chile	Instituto de Salud Publica de Chile
EPI1574885	NA	Chile	2019-Jul-14	B/Santiago/64840/2019	Instituto de Salud Publica de Chile	Instituto de Salud Publica de Chile
EPI368778	NA	Chile	2012-Feb-19	B/Santiago/11896/2012	Instituto de Salud Publica de Chile	Centers for Disease Control and Prevention
EPI378194	NA	Chile	2012-May-29	B/Santiago/38204/2012	Instituto de Salud Publica de Chile	Centers for Disease Control and Prevention
EPI452255	NA	Chile	2013-Apr-09	B/Santiago/23499/2013	Instituto de Salud Publica de Chile	Centers for Disease Control and Prevention
EPI504554	NA	Chile	2013-Dec-06	B/Santiago/80686/2013	Instituto de Salud Publica de Chile	Centers for Disease Control and Prevention
EPI569115	NA	Chile	2014-Nov-01	B/Santiago/71933/2014	Instituto de Salud Publica de Chile	Centers for Disease Control and Prevention
EPI569127	NA	Chile	2014-Nov-08	B/Santiago/73755/2014	Instituto de Salud Publica de Chile	Centers for Disease Control and Prevention
EPI695100	NA	Chile	2015-Oct-22	B/Santiago/75552/2015	Instituto de Salud Publica de Chile	Centers for Disease Control and Prevention
EPI695116	NA	Chile	2015-Oct-17	B/Pucon/75541/2015	Instituto de Salud Publica de Chile	Centers for Disease Control and Prevention
EPI807426	NA	Chile	2016-Mar-16	B/Santiago/20269/2016	Instituto de Salud Publica de Chile	Centers for Disease Control and Prevention
EPI830816	NA	Chile	2016-Jun-13	B/Valparaiso/49570/2016	Instituto de Salud Publica de Chile	Centers for Disease Control and Prevention
EPI1137836	NA	Chile	2017-Oct-02	B/Santiago/75265/2017	Instituto de Salud Publica de Chile	Centers for Disease Control and Prevention
EPI1137972	NA	Chile	2017-Oct-25	B/Santiago/81374/2017	Instituto de Salud Publica de Chile	Centers for Disease Control and Prevention
EPI1261286	NA	Chile	2018-Apr-23	B/Santiago/34517/2018	Instituto de Salud Publica de Chile	Centers for Disease Control and Prevention
EPI1320605	NA	Chile	2018-Jun-27	B/Santiago/53060/2018	Instituto de Salud Publica de Chile	Centers for Disease Control and Prevention
EPI482836	NA	Paraguay	2013-Jun-27	B/Paraguay/022/2013	Central Laboratory of Public Health	Centers for Disease Control and Prevention
EPI544223	NA	Paraguay	2014-Jun-24	B/Paraguay/0068/2014	Central Laboratory of Public Health	Centers for Disease Control and Prevention
EPI562678	NA	Paraguay	2014-Nov-25	B/Paraguay/2429/2014	Central Laboratory of Public Health	Centers for Disease Control and Prevention
EPI708897	NA	Paraguay	2015-Aug-05	B/Paraguay/6712/2015	Central Laboratory of Public Health	Centers for Disease Control and Prevention
EPI753833	NA	Paraguay	2015-Aug-31	B/Paraguay/0905/2015	Central Laboratory of Public Health	Centers for Disease Control and Prevention
EPI830606	NA	Paraguay	2016-Mar-29	B/Paraguay/3296/2016	Central Laboratory of Public Health	Centers for Disease Control and Prevention
EPI892125	NA	Paraguay	2016-Nov-18	B/Paraguay/5243/2016	Central Laboratory of Public Health	Centers for Disease Control and Prevention
EPI1078993	NA	Paraguay	2017-Feb-19	B/Paraguay/3099/2017	Central Laboratory of Public Health	Centers for Disease Control and Prevention
EPI1079017	NA	Paraguay	2017-May-09	B/Paraguay/8072/2017	Central Laboratory of Public Health	Centers for Disease Control and Prevention
EPI1278358	NA	Paraguay	2018-Apr-17	B/Paraguay/0336/2018	Central Laboratory of Public Health	Centers for Disease Control and Prevention
EPI1278414	NA	Paraguay	2018-Jun-18	B/Paraguay/6288/2018	Central Laboratory of Public Health	Centers for Disease Control and Prevention
EPI397175	NA	Uruguay	2012-May-14	B/Uruguay/153/2012	Departamento de Laboratorio de Salud Pública (DLSP)	Centers for Disease Control and Prevention
EPI397495	NA	Uruguay	2012-Jul-19	B/Uruguay/358/2012	Departamento de Laboratorio de Salud Pública (DLSP)	Centers for Disease Control and Prevention
EPI543635	NA	Uruguay	2014-Jun-02	B/Uruguay/143/2014	Departamento de Laboratorio de Salud Pública (DLSP)	Centers for Disease Control and Prevention
EPI1034132	NA	Uruguay	2017-May-23	B/Uruguay/106/2017	Departamento de Laboratorio de Salud Pública (DLSP)	Centers for Disease Control and Prevention
EPI1034140	NA	Uruguay	2017-May-25	B/Uruguay/113/2017	Departamento de Laboratorio de Salud Pública (DLSP)	Centers for Disease Control and Prevention
EPI1034147	NA	Uruguay	2017-May-26	B/Uruguay/119/2017	Departamento de Laboratorio de Salud Pública (DLSP)	Centers for Disease Control and Prevention
EPI1034163	NA	Uruguay	2017-May-29	B/Uruguay/126/2017	Departamento de Laboratorio de Salud Pública (DLSP)	Centers for Disease Control and Prevention
EPI1053030	NA	Uruguay	2017-Jun-20	B/Uruguay/403/2017	Departamento de Laboratorio de Salud Pública (DLSP)	Centers for Disease Control and Prevention
EPI1053038	NA	Uruguay	2017-Jun-30	B/Uruguay/527/2017	Departamento de Laboratorio de Salud Pública (DLSP)	Centers for Disease Control and Prevention
EPI1053046	NA	Uruguay	2017-Jun-15	B/Uruguay/338/2017	Departamento de Laboratorio de Salud Pública (DLSP)	Centers for Disease Control and Prevention
EPI1312795	NA	Uruguay	2018-Jul-24	B/Uruguay/571/2018	Departamento de Laboratorio de Salud Pública (DLSP)	Centers for Disease Control and Prevention
EPI1312803	NA	Uruguay	2018-Jul-19	B/Uruguay/553/2018	Departamento de Laboratorio de Salud Pública (DLSP)	Centers for Disease Control and Prevention
EPI1312811	NA	Uruguay	2018-Feb-20	B/Uruguay/033/2018	Departamento de Laboratorio de Salud Pública (DLSP)	Centers for Disease Control and Prevention
EPI1694429	NA	Uruguay	2012-Aug-18	B/Uruguay/488/2012	Departamento de Laboratorio de Salud Pública (DLSP)	National Influenza Center, Ministry of Health
EPI1694438	NA	Uruguay	2012-Feb-07	B/Uruguay/252/2012	Departamento de Laboratorio de Salud Pública (DLSP)	National Influenza Center, Ministry of Health
EPI1694440	NA	Uruguay	2012-Feb-07	B/Uruguay/266/2012	Departamento de Laboratorio de Salud Pública (DLSP)	National Influenza Center, Ministry of Health
EPI1694442	NA	Uruguay	2012-Apr-07	B/Uruguay/276/2012	Departamento de Laboratorio de Salud Pública (DLSP)	National Influenza Center, Ministry of Health
EPI1694444	NA	Uruguay	2012-Aug-30	B/Uruguay/515/2012	Departamento de Laboratorio de Salud Pública (DLSP)	National Influenza Center, Ministry of Health
EPI1694452	NA	Uruguay	2014-Aug-08	B/Uruguay/422/2014	Departamento de Laboratorio de Salud Pública (DLSP)	National Influenza Center, Ministry of Health
EPI1694457	NA	Uruguay	2015-Nov-27	B/Uruguay/372/2015	Departamento de Laboratorio de Salud Pública (DLSP)	National Influenza Center, Ministry of Health
EPI1694459	NA	Uruguay	2015-Oct-23	B/Uruguay/309/2015	Departamento de Laboratorio de Salud Pública (DLSP)	National Influenza Center, Ministry of Health
EPI1694496	NA	Uruguay	2018-Sep-18	B/Uruguay/1054/2018	Departamento de Laboratorio de Salud Pública (DLSP)	National Influenza Center, Ministry of Health
EPI1694498	NA	Uruguay	2018-Sep-12	B/Uruguay/1005/2018	Departamento de Laboratorio de Salud Pública (DLSP)	National Influenza Center, Ministry of Health
EPI1694500	NA	Uruguay	2018-Aug-22	B/Uruguay/816/2018	Departamento de Laboratorio de Salud Pública (DLSP)	National Influenza Center, Ministry of Health
EPI1694502	NA	Uruguay	2018-Aug-06	B/Uruguay/727/2018	Departamento de Laboratorio de Salud Pública (DLSP)	National Influenza Center, Ministry of Health
EPI1694505	NA	Uruguay	2018-Sep-21	B/Uruguay/1070/2018	Departamento de Laboratorio de Salud Pública (DLSP)	National Influenza Center, Ministry of Health

Table S1. Names and accession numbers for the samples used in this study. The Uruguayan sequences generated have been deposited in the GISAID database.

	52	58	87	106	117	121	129	144	146	162	163	164	169	175	180	252
B/Brisbane/60/2008	**K**	**L**	**V**	**Q**	**I**	**T**	**N**	**P**	**I**	**K**	**N**	**D**	**A**	**I**	**I**	**V**
B/SaoPaulo/1187418IAL/2019	.	.	.	.	V	.	G	.	.	del	del	.	.	.	V	.
B/SaoPaulo/1211465IAL/2019	.	.	.	.	V	.	G	.	.	del	del	.	.	.	V	.
**B/Uruguay/748/2019**	.	.	.	.	V	.	G	.	.	del	del	.	.	.	V	.
**B/Uruguay/708/2019**	.	.	.	.	V	.	G	.	.	del	del	.	.	.	V	.
**B/Uruguay/753/2019**	.	.	.	.	V	.	G	.	.	del	del	.	.	.	V	.
B/Paraguay/0620/2018	.	.	.	.	V	.	G	.	.	del	del	.	.	.	V	M
B/Bahia/735/2019	.	.	.	.	V	.	G	.	.	del	del	.	.	.	V	.
B/Brazil/4835/2018	.	.	.	.	V	.	G	.	.	del	del	.	.	.	V	.
B/Brazil/2470/2018	.	.	.	.	V	.	G	.	.	del	del	.	.	.	V	.
B/Bahia/148/2019	.	.	.	.	V	.	G	.	.	del	del	.	.	.	V	.
B/Argentina/9/2018	.	.	.	.	V	.	G	.	.	del	del	.	.	.	V	.
B/Argentina/13738/2018	.	.	.	.	V	.	G	.	.	del	del	.	.	.	V	.
B/Talca/63221/2019	.	.	.	.	V	.	G	.	.	del	del	.	.	.	V	.
B/Santiago/62161/2019	.	.	.	.	V	.	G	.	.	del	del	.	.	.	V	.
B/Argentina/2110/2017	.	.	.	.	V	.	G	.	.	del	del	.	.	.	V	.
B/Argentina/13339/2017	.	.	.	.	V	.	G	.	.	del	del	.	.	.	V	.
B/Santiago/54067/2017	.	.	.	.	V	.	G	.	.	del	del	.	.	.	V	.
B/Santiago/51375/2018	.	.	.	.	V	.	G	.	.	del	del	.	.	.	V	.
B/Santiago/34511/2018	.	.	.	.	V	.	G	.	.	del	del	.	.	.	V	.
B/Paraguay/7301/2018	.	.	.	.	V	.	G	.	.	del	del	.	.	.	V	.
B/Colorado/06/2017	.	.	.	.	V	.	G	.	.	del	del	.	.	.	V	.
B/Santiago/25385/2017	.	.	.	.	V	.	D	.	.	.	.	.	.	.	.	.
B/Washington/02/2019	.	.	.	.	V	.	D	.		del	del	del	.	.	.	.
**B/Uruguay/789/2016**	.	.	.	.	V	.	D	.	.	.	.	.	.	.	.	.
B/Santiago/45998/2016	.	.	.	.	V	.	D	.	.	.	.	.	.	.	.	.
B/Brazil/80287/2015	.	.	.	.	V	.	D	.	.	.	.	.	.	.	.	.
B/Brazil/6128/2017	.	.	.	.	V	.	D	.	.	.	.	.	.	.	.	.
B/Paraguay/5894/2016	.	.	.	.	V	.	D	.	.	.	.	.	.	.	.	.
B/Paraguay/2377/2016	.	.	.	.	V	.	D	.	.	.	.	.	.	.	.	.
B/Brazil/0496/2016	.	.	.	.	V	.	D	.	.	.	.	.	.	.	.	.
B/Ceara/144952-IEC/2016	.	.	.	.	V	.	D	.	.	.	.	.	.	.	.	.
B/Santiago/44313/2016	.	.	.	.	V	.	D	.	.	.	.	.	.	.	.	.
B/Brazil/4558/2017	.	.	.	.	V	.	D	.	.	.	.	.	.	.	.	.
**B/Uruguay/137/2017**	.	.	A	.	V	.	D	.	.	.	.	.	.	V	.	.
**B/Uruguay/138/2017**	.	.	A	.	V	.	D	.	.	.	.	.	.	V	.	.
**B/Uruguay/140/2017**	.	.	A	.	V	.	D	.	.	.	.	.	.	V	.	.
**B/Uruguay/511/2017**	.	.	A	.	V	.	D	.	.	.	.	.	.	V	.	.
**B/Uruguay/379/2017**	.	.	A	.	V	.	D	.	.	.	.	.	.	V	.	.
**B/Uruguay/383/2017**	.	.	A	.	V	.	D	.	.	.	.	.	.	V	.	.
**B/Uruguay/830/2017**	.	.	A	.	V	.	D	.	.	.	.	.	.	V	.	.
**B/Uruguay/659/2017**	.	.	A	.	V	.	D	.	.	.	.	.	.	V	.	.
**B/Uruguay/700/2017**	.	.	A	.	V	.	D	.	.	.	.	.	.	V	.	.
B/Santiago/80460/2015	.	.	.	.	V	.	D	.	.	.	.	.	.	.	.	M
B/Paraguay/8037/2017	.	.	.	.	V	.	D	.	.	.	.	.	.	.	.	M
B/Argentina/268/2016	.	.	.	.	V	.	D	.	.	.	.	.	.	.	.	M
**B/Uruguay/934/2016**	.	.	.	.	V	N	D	.	.	.	.	.	.	.	.	M
B/Florida/78/2015	.	.	.	.	V	.	D	.	.	.	.	.	.	.	.	.
B/Argentina/11461/2015	.	.	.	.	V	.	D	.	.	.	.	.	.	.	.	.
B/Brazil/6297/2015	.	.	.	.	V	.	D	.	.	.	.	.	.	.	.	.
**B/Uruguay/124/2017**	.	.	.	.	V	.	D	.	.	.	.	.	.	.	.	.
**B/Uruguay/98/2015**	.	.	.	.	.	.	D	.	.	.	.	.	.	.	.	.
**B/Uruguay/102/2015**	.	.	.	.	.	.	D	.	.	.	.	.	.	.	.	.
**B/Uruguay/02/2016**	.	.	.	.	.	.	D	.	.	.	.	.	.	.	.	.
**B/Uruguay/335/2015**	.	.	.	.	.	.	D	.	.	.	.	.	.	.	.	.
**B/Uruguay/345/2015**	.	.	.	R	.	.	D	.	.	.	.	.	.	.	.	.
**B/Uruguay/289/2015**	.	.	.	.	.	.	D	.	.	.	.	.	.	.	.	.
**B/Uruguay/384/2015**	.	.	.	.	.	.	D	.	.	.	.	.	.	.	.	.
**B/Uruguay/105/2015**	.	.	.	.	.	.	D	.	.	.	.	.	.	.	.	.
**B/Uruguay/396/2015**	.	.	.	.	.	.	D	.	.	.	.	.	.	.	.	.
B/BuenosAires/10986152/2014	.	.	.	.	.	.	D	.	V	.	.	.	.	.	.	.
B/Paraguay/4674/2014	.	.	.	.	.	.	D	.	V	.	.	.	.	.	.	.
B/Paraguay/5133/2015	.	.	.	.	.	.	D	.	V	.	.	.	.	.	.	.
B/Valparaiso/81181/2015	.	.	.	.	.	.	D	.	V	.	.	.	.	.	.	.
B/Brazil/5137/2014	.	.	.	.	.	.	D	.	V	.	.	.	.	.	.	.
B/Paraguay/1131/2015	.	.	.	.	.	.	D	.	V	.	.	.	.	.	.	.
B/Concepcion/264/2014	.	.	.	.	.	.	D	.	V	.	.	.	.	.	.	.
B/Argentina/154/2014	.	.	.	.	.	.	D	.	V	.	.	.	.	.	.	.
B/PuertoMontt/81199/2013	.	.	.	.	.	.	D	.	V	.	.	.	.	.	.	.
B/Brazil/2920/2014	.	.	.	.	.	.	D	.	V	.	.	.	.	.	.	.
B/Santiago/69223/2014	.	.	.	.	.	.	D	.	V	.	.	.	.	.	.	.
B/Santiago/82832/2013	.	.	.	.	.	.	G	.	V	.	.	.	.	.	.	.
**B/Uruguay/177/2013**	.	.	.	.	.	.	D	.	V	.	.	.	.	.	.	.
B/BuenosAires/770/2012	.	.	.	.	.	.	.	.	V	.	.	.	.	.	.	.
B/Brazil/6004/2013	.	.	.	.	.	A	.	.	V	.	D	.	.	.	.	.
B/Santiago/34960/2012	.	.	.	.	.	.	.	.	V	.	.	.	.	.	.	.
B/Santiago/49303/2012	.	.	.	.	.	.	.	.	V	.	.	.	.	.	.	.
**B/Uruguay/312/2012**	.	.	.	.	.	.	.	.	V	.	.	.	.	.	.	.
**B/Uruguay/257/2012**	.	.	.	.	.	.	.	L	V	.	.	.	.	.	.	.
B/Brazil/3742/2013	.	.	.	.	.	.	.	.	V	.	.	.	E	.	.	.
B/Formosa/V2367/2012	.	.	.	.	.	.	.	.	V	.	.	.	E	.	.	.
B/Paraguay/672/2014	.	.	.	.	.	.	.	.	V	.	.	.	E	.	.	.
**B/Uruguay/406/2014**	.	.	.	.	.	.	.	.	V	.	.	.	E	.	.	.
**B/Uruguay/864/2013**	.	.	.	.	.	.	.	.	V	.	.	.	E	.	.	.
**B/Uruguay/578/2014**	.	.	.	.	.	.	.	.	V	.	.	.	E	.	.	.
**B/Uruguay/94/2015**	.	.	.	.	.	.	.	.	V	.	.	.	E	.	.	.
B/Brazil/0944/2012	.	.	.	.	.	.	.	.	V	.	.	.	.	.	.	.
B/Paraguay/190/2012	.	.	.	.	.	.	.	.	V	.	.	.	.	.	.	.
B/Paraguay/252/2012	.	.	.	.	.	.	.	.	V	.	.	.	.	.	.	.
**B/Uruguay/236/2012**	R	.	.	.	.	.	.	.	V	.	.	.	.	.	.	.
**B/Uruguay/311/2012**	R	.	.	.	.	.	.	.	V	.	.	.	.	.	.	.
B/Montana/05/2012	.	P	I	.	.	.	S	.	V	.	.	.	.	.	.	.
B/Victoria/304/2006	.	.	.	.	.	.	.	.	V	.	.	.	.	.	.	.
B/Argentina/R158/2010	.	.	.	.	.	.	.	.	V	.	.	.	.	.	.	.
B/Uruguay/12/2008	.	.	.	.	.	.	.	.	V	.	.	.	.	.	.	.
B/Fujian/Gulou1272/2008	.	.	.	.		A	.	.	V	.	.	.	.	.	.	.
B/Malaysia/2506/2004	.	.	.	.	.	.	.	.	V	.	.	.	.	.	.	.
B/Singapore/19/2009	.	.	.	.	.	.	.	.	V	.	.	.	.	.	.	.
B/Victoria/2/87	.	.	.	.	.	.	T	.	V	.	.	.	.	V	.	.

Table S2. Comparison of the amino acid sequence analysis of the HA1 subunit of the Influenza B/Victoria viruses from 2012-2019 with B/Brisbane/60/2008 reference strain. A dot (.) denotes same amino acid at the same position in the reference sequence.

	48	83	88	108	116	124	150	165	172	181	188	198	202	229	232	251	298	312
B/Phuket/3073/2013	**R**	**I**	**R**	**P**	**K**	**V**	**I**	**Y**	**L**	**T**	**T**	**T**	**S**	**D**	**D**	**M**	**E**	**K**
B/Santiago/64801/2019	.	.	.	.	.	.	.	.	Q	.	.	.	.	.	N	V	.	.
B/Santiago/64840/2019	.	.	.	.	.	.	.	.	Q	.	.	.	.	.	N	V	.	.
**B/Uruguay/727/2018**	.	.	.	.	.	.	.	.	Q	.	.	.	.	.	N	V	.	.
**B/Uruguay/816/2018**	.	.	.	.	.	.	.	.	Q	.	.	.	.	.	N	V	.	.
**B/Uruguay/1005/2018**	.	.	.	.	.	.	.	.	Q	.	.	.	.	.	N	V	.	.
B/Argentina/13785/2018	.	.	.	.	.	.	.	.	Q	.	.	.	.	.	N	V	.	.
B/Brazil/7103/2018	.	.	.	.	.	.	.	.	Q	.	.	.	.	.	N	V	.	.
**B/Uruguay/1054/2018**	.	.	.	.	.	.	.	.	Q	.	.	.	.	.	N	V	.	.
**B/Uruguay/033/2018**	.	.	.	.	.	.	.	.	Q	.	.	.	.	.	N	V	.	.
**B/Uruguay/1070/2018**	.	.	.	.	.	.	V	.	Q	.	.	.	.	N	.	V	.	.
**B/Uruguay/553/2018**	.	.	.	.	.	.	.	.	Q	.	.	.	.	N	.	V	.	.
**B/Uruguay/626/2018**	.	.	.	.	.	.	.	.	Q	.	.	.	.	N	.	V	.	.
**B/Uruguay/899/2018**	.	.	.	.	.	.	.	.	Q	.	.	.	.	N	.	V	.	.
**B/Uruguay/835/2018**	.	.	.	.	.	.	.	.	Q	.	.	.	.	N	.	V	.	.
**B/Uruguay/571/2018**	.	.	.	.	.	.	.	.	Q	.	.	.	.	.	.	V	.	.
B/Brazil/0379/2018	.	.	.	.	.	.	.	.	Q	.	.	.	.	.	.	V	.	.
B/Argentina/14/2018	.	.	.	.	.	.	.	.	Q	.	.	.	.	.	.	V	.	.
B/Santiago/53060/2018	.	.	.	.	.	.	.	.	Q	.	.	.	.	.	N	V	.	.
B/Santiago/75265/2017	.	.	.	.	.	.	.	.	Q	.	.	.	.	.	.	V	.	.
B/Santiago/34517/2018	.	.	.	.	.	.	.	.	Q	.	.	.	.	.	.	V	.	.
B/Argentina/1744/2017	.	.	.	.	.	.	.	.	Q	.	.	.	.	.	.	V	.	.
B/Argentina/13252/2017	.	.	.	.	.	.	.	.	Q	.	.	.	.	.	.	V	.	.
**B/Uruguay/1064/2017**	.	.	.	.	.	.	.	.	Q	.	.	.	.	.	.	V	.	.
B/Paraguay/3099/2017	.	.	.	.	.	.	.	.	Q	.	.	.	.	.	.	V	.	.
B/Paraguay/8072/2017	.	.	.	.	.	.	.	.	Q	.	.	.	.	.	.	V	.	.
**B/Uruguay/106/2017**	.	.	.	.	.	.	.	.	Q	.	.	.	.	.	.	V	.	.
**B/Uruguay/113/2017**	.	.	.	.	.	.	.	.	Q	.	.	.	.	.	.	V	.	.
**B/Uruguay/527/2017**	.	.	.	.	.	.	.	.	Q	.	.	.	.	.	.	V	.	.
**B/Uruguay/126/2017**	.	.	.	.	.	.	.	.	Q	.	.	.	.	.	.	V	.	.
B/Paraguay/6288/2018	.	.	.	.	.	.	.	.	Q	.	.	.	.	.	.	V	.	.
B/Paraguay/0336/2018	.	.	.	.	.	.	.	.	Q	.	.	.	.	.	.	V	.	.
B/Santiago/81374/2017	.	.	.	.	.	.	.	.	Q	.						V	.	.
**B/Uruguay/338/2017**	.	.	.	.	.	.	.	.	Q	.	.	.	.	.	.	V	.	.
**B/Uruguay/119/2017**	.	.	.	.	.	.	.	.	Q	.	.	.	.	.	.	V	.	.
**B/Uruguay/403/2017**	.	.	.	.	.	.	.	.	Q	.	.	.	.	.	.	V	.	.
B/Paraguay/3296/2016	.	.	.	.	.	.	.	.	Q	.	.	.	.	.	.	V	.	.
B/Pucon/75541/2015	.	.	.	.	.	.	.	.	Q	.	.	.	.	.	.	V	.	.
B/Paraguay/0905/2015	.	.	.	.	.	.	.	.	Q	.	.	.	.	.	.	V	.	.
**B/Uruguay/309/2015**	.	.	.	.	.	.	.	.	Q	.	.	.	.	.	.	V	.	.
**B/Uruguay/372/2015**	.	.	.	.	.	.	.	.	Q	.	.	.	.	.	.	V	.	.
B/Brazil/0589/2015	.	.	.	.	.	.	.	.	Q	.	.	.	.	.	.	V	.	.
B/Pernambuco/144718IEC/2016	.	.	.	.	.	.	.	.	Q	.	.	.	.	.	.	V	.	.
B/Argentina/11616/2015	.	.	.	.	.	.	.	.	Q	.	.	.	.	.	.	V	.	.
B/Argentina/19/2016	.	.	.	.	.	.	.	.	Q	.	.	.	.	.	.	V	.	.
B/Santiago/75552/2015	.	.	.	.	.	.	.	.	Q	.	.	.	.	.	N	V	.	.
B/Paraguay/6712/2015	.	.	.	.	.	.	.	.	Q	.	.	.	.	.	.	V	.	.
B/Valparaiso/49570/2016	.	.	.	.	.	.	.	.	Q	.	.	.	.	.	.	V	.	.
B/Santiago/20269/2016	.	.	.	.	.	.	.	.	Q	.	.	.	.	.	.	V	.	.
B/Brazil/8134/2017	.	.	.	.	.	.	.	.	Q	.	.	.	.	.	.	V	.	.
B/Argentina/220/2016	.	.	.	.	.	.	.	.	Q	.	.	.	.	.	.	V	.	.
B/Paraguay/5243/2016	.	.	.	.	.	.	V	.	Q	.	.	.	.	.	.	V	.	.
B/Brazil/3889/2017	.	.	.	.	.	.	V	.	Q	.	.	.	.	.	.	V	.	.
B/Santiago/73755/2014	.	.	.	.	.	.	.	.	Q	.	.	.	.	.	.	.	.	.
B/Brazil/36401/2015	.	.	.	.	.	.	.	.	Q	.	.	.	.	.	.	.	.	.
B/Santiago/71933/2014	.	.	.	.	.	.	.	.	Q	.	.	.	.	.	.	.	.	.
B/Brazil/1716/2014	.	.	.	.	.	.	.	.	Q	.	.	.	.	.	.	.	.	.
B/Brazil/2282/2014	.	.	.	.	.	.	.	.	Q	.	.	.	.	.	.	.	.	.
**B/Uruguay/143/2014**	.	V	.	.	.	.	.	.	Q	.	.	.	.	.	.	.	.	.
B/SantaFe/1149/2014	.	.	.	.	.	.	.	.	Q	.	.	.	.	.	.	.	.	.
B/Paraguay/2429/2014	.	.	.	.	.	.	.	.	Q	.	.	.	.	.	.	.	.	.
B/RioGrandeDoNorte/140088/2016	.	.	.	.	.	.	.	.	Q	.	.	.	.	.	.	I	.	.
**B/Uruguay/422/2014**	.	.	.	.	.	.	.	.	Q	.	.	.	.	.	.	.	.	.
B/GuangdongLiwan/1133/2014	.	.	.	.	.	.	.	.	.	.	.	.	.	G	.	.	.	.
B/Wisconsin/01/2010	.	.	.	.	N	.	.	.	.	.	.	.	.	.	.	.	K	E
B/Santiago/80686/2013	.	.	.	.	N	.	.	.	.	K	.	.	N	.	.	.	.	.
B/Natal/119888/2012	.	.	.	.	N	.	.	.	.	K	.	.	N	.	.	.	.	.
B/Brazil/325/2013	.	.	.	.	N	.	.	.	.	K	.	.	N	.	.	.	.	.
B/Paraguay/022/2013	.	.	.	.	N	.	.	.	.	K	.	.	N	.	.	.	.	.
B/Paraguay/0068/2014	.	.	.	.	N	.	.	.	.	K	.	.	N	.	.	.	.	.
B/Santiago/11896/2012	.	.	.	.	N	A	.	.	Q	.	.	.	N	.	.	V	K	E
B/Santiago/38204/2012	K	.	.	A	N	.	S	N	.	A	.	.	N	G	.	.	K	E
B/Massachusetts/02/2012	K	.	.	A	N	.	S	N	.	A	.	I	N	G	.	.	K	E
**B/Uruguay/276/2012**	K	.	.	A	N	.	S	N	.	A	.	.	N	G	.	.	K	E
B/SantaFe/1079/2014	K	.	.	A	N	.	S	N	.	A	.	.	N	G	.	.	K	E
B/Tucuman/A285/2012	K	.	.	A	N	.	S	N	.	A	.	.	N	G	.	.	K	E
B/BuenosAires/8833/2012	K	.	.	A	N	.	S	N	.	A	.	.	N	G	.	.	K	E
**B/Uruguay/153/2012**	K	.	.	A	N	.	S	N	.	A	.	.	N	G	.	.	K	E
**B/Uruguay/252/2012**	K	.	.	A	N	.	S	N	.	A	.	.	N	G	.	.	K	E
**B/Uruguay/488/2012**	K	.	.	A	N	.	S	N	.	A	.	.	N	G	.	.	K	E
**B/Uruguay/515/2012**	K	.	.	A	N	A	S	N	.	A	.	.	N	G	.	.	K	E
B/Brazil/374/2013	K	.	.	A	N	.	S	N	.	A	A	.	N	G	.	.	K	E
B/Santiago/23499/2013	K	.	.	A	N	.	S	N	.	A	N	.	N	G	.	.	K	E
**B/Uruguay/358/2012**	K	.	.	A	N	.	S	N	.	A	.	.	N	G	.	.	K	E
**B/Uruguay/266/2012**	K	.	.	A	N	.	S	N	.	A	.	.	N	G	.	.	K	E
B/Florida/4/2006	.	.	K	.	N	.	S	N	.	.	.	.	N	S	.	.	K	E
B/Yamagata/16/88	K	.	.	.	N	.	N	X	.	.	.	.	N	.	N	V	K	E

Table S3. Comparison of the amino acid sequence analysis of the HA1 subunit of the Influenza B/Yamagata viruses from 2012-2019 with B/Phuket/3013/2013 reference strain. A dot (.) denotes same amino acid at the same position in the reference sequence.

	45	46	51	59	60	68	70	71	72	73	74	75	106	120	125	157	198	220	233	235	290	295	303	315	340	342	345	358	371	384	395	401	403
B/Brisbane/60/2008	**I**	**T**	**P**	**N**	**V**	**T**	**G**	**V**	**T**	**L**	**L**	**L**	**T**	**I**	**N**	**I**	**N**	**K**	**G**	**N**	**A**	**S**	**V**	**R**	**N**	**D**	**S**	**E**	**K**	**D**	**A**	**V**	**M**
B/SaoPaulo/1190756-IAL/2019	.	.	.	.	.	.	.	.	.	.	.	.	.	V	.	.	.	N	.	.	.	R	.	.	D	.	.	K	Q	G	D	.	.
B/SaoPaulo/1187418-IAL/2019	.	.	.	.	.	.	.	.	.	.	.	.	.	V	.	.	.	N	.	.	.	R	.	.	D	.	.	K	Q	G	D	.	.
B/SaoPaulo/1211465-IAL/2019	.	.	.	.	.	.	.	.	.	.	.	.	.	V	.	.	.	N	.	.	.	R	.	.	D	.	.	K	Q	G	D	.	.
B/Paraguay/0620/2018	.	.	.	.	.	.	.	.	A	.	.	.	.	V	.	.	.	N	.	.	.	R	.	.	D	.	.	K	Q	G	D	.	.
B/Brazil/4835/2018	.	.	.	.	.	.	.	.	..	.	.	.	.	V	.	.	.	N	.	.	.	R	.	.	D	.	.	K	Q	G	D	.	.
B/Bahia/735/2019	.	.	.	.	.	.	.	.	.	.	.	.	.	V	.	.	.	N	.	.	.	R	.	.	D	.	N	K	Q	G	D	.	.
B/Argentina/9/2018	.	.	.	.	.	.	.	.	.	.	.	.	.	V	.	.	.	N	.	.	.	R	.	.	D	.	.	K	Q	G	.	.	.
**B/Uruguay/708/2019**	.	.	.	.	.	.	.	.	.	.	.	.	.	V	.	.	.	N	.	.	.	R	.	.	D	.	.	K	Q	G	D	.	.
**B/Uruguay/748/2019**	.	.	.	.	.	.	.	.	.	.	.	.	.	V	.	.	.	N	.	.	.	R	.	.	D	.	.	K	Q	G	D	.	.
**B/Uruguay/753/2019**	-	-	-	-	-	-	-	-	-	-	-	-	.	V	.	.	.	N	.	.	.	R	.	.	D	.	.	K	Q	G	D	.	.
B/Santiago/54067/2017	.	.	.	.	.	.	.	.	.	.	.	.	.	V	.	.	.	N	.	.	.	R	.	.	D	.	.	K	Q	G	.	.	.
B/Santiago/51375/2018	.	.	.	.	.	.	.	.	.	.	.	.	.	.	.	.	.	N	.	.	.	R	.	.	D	.	.	K	Q	G	V	.	I
B/Santiago/34511/2018	.	.	.	.	.	.	.	.	.	.	.	.	.	.	.	.	.	N	.	.	.	R	.	.	D	.	.	K	Q	G	V	.	.
B/Colorado/06/2017	.	.	.	.	.	.	.	.	.	.	.	.	.	V	.	.	.	N	.	.	.	R	.	.	D	.	.	K	Q	G	.	.	.
B/Parana/115/2019	.	.	.	.	.	.	E	.	.	.	.	.	.	V	.	.	.	N	.	.	.	R	.	.	D	.	.	K	Q	G	.	.	.
B/Bahia/148/2019	.	.	.	.	.	.	.	.	.	.	.	.	.	V	.	.	.	N	.	.	.	R	.	.	D	.	.	K	Q	G	.	.	.
B/Santiago/62161/2019	.	.	.	.	.	.	.	.	.	.	.	.	.	V	.	.	.	N	.	.	.	R	.	.	D	.	.	K	Q	G	.	.	.
B/Talca/63221/2019	.	.	.	.	.	.	.	.	.	.	.	.	.	V	.	.	.	N	.	.	.	R	.	.	D	.	.	K	Q	G	.	.	.
B/Argentina/2110/2017	.	.	.	.	.	.	.	.	.	.	.	.	.	V	.	.	.	N	.	.	.	R	.	.	D	.	.	K	Q	G	.	.	.
B/Parana/206/2019	.	.	.	.	.	.	.	.	.	.	I	.	.	V	.	.	.	N	.	.	.	R	.	.	D	.	.	K	Q	G	.	.	.
B/Brazil/2470/2018	.	.	.	.	.	.	.	.	.	.	.	.	.	V	.	.	.	N	.	.	.	R	.	.	D	.	.	K	Q	G	.	.	.
B/Argentina/13738/2018	.	.	.	.	.	.	.	.	.	.	.	.	.	V	.	.	.	N	.	.	.	R	.	.	D	.	.	K	Q	G	.	.	.
B/Argentina/13339/2017	.	.	.	.	.	.	.	.	.	.	.	.	.	V	.	.	.	N	.	.	.	R	.	.	D	.	.	K	Q	G	.	.	.
B/Paraguay/7301/2018	.	.	.	.	.	.	.	.	.	.	.	.	.	V	.	.	.	N	.	.	.	R	.	.	D	.	.	K	Q	G	.	.	.
B/Brazil/6128/2017	.	.	.	.	.	.	.	.	.	F	.	.	.	V	.	.	.	N	.	.	.	R	.	.	D	.	.	K	.	G	.	.	.
B/Ceara/144952IEC/2016	.	.	.	.	.	.	.	.	.	.	.	.	.	V	.	.	.	N	.	.	.	R	.	.	D	.	.	K	.	G	.	.	.
**B/Uruguay/830/2017**	-	-	-	.	.	A	.	.	.	.	.	.	I	V	.	.	.	N	.	.	.	R	.	.	D	.	.	K	.	G	.	I	.
**B/Uruguay/700/2017**	-	-	-	.	.	A	.	.	.	.	.	.	I	V	.	.	.	N	.	.	.	R	.	.	D	.	.	K	.	G	.	I	.
**B/Uruguay/137/2017**	.	.	.	.	.	A	.	.	.	.	.	.	I	V	.	.	.	N	.	.	.	R	.	.	D	.	.	K	.	G	.	I	.
**B/Uruguay/138/2017**	.	.	.	.	.	A	.	.	.	.	.	.	I	V	.	.	.	N	.	.	.	R	.	.	D	.	.	K	.	G	.	I	.
**B/Uruguay/140/2017**	.	.	.	.	.	A	.	.	.	.	I	.	I	V	.	.	.	N	.	.	.	R	.	.	D	.	.	K	.	G	.	I	.
**B/Uruguay/511/2017**	.	.	.	.	.	A	.	.	.	.	.	.	I	V	.	.	.	N	.	.	.	R	.	.	D	.	.	K	.	G	.	I	.
**B/Uruguay/379/2017**	.	.	.	.	.	A	.	.	.	.	.	.	I	V	.	.	.	N	.	.	.	R	.	.	D	.	.	K	.	G	.	I	.
**B/Uruguay/383/2017**	.	.	.	.	.	A	.	.	.	.	.	.	I	V	.	.	.	N	.	.	.	R	.	.	D	.	.	K	.	G	.	I	.
**B/Uruguay/673/2017**	-	-	-	-	I	A	.	.	.	.	.	F	I	V	.	.	.	N	.	.	.	R	.	.	D	.	.	K	.	G	.	I	.
**B/Uruguay/789/2016**	-	-	-	.	.	.	.	.	.	.	.	.	.	V	.	V	.	N	.	.	V	R	.	.	D	.	.	K	.	G	.	.	.
B/Washington/02/2019	.	.	.	.	.	.	.	.	.	.	.	.	.	V	.	.	.	N	.	.	.	R	.	.	D	.	.	K	.	G	T	.	.
B/Santiago/44313/2016	.	.	.	.	.	.	.	.	.	.	.	.	.	V	.	.	.	N	.	.	.	R	.	.	D	.	.	K	.	G	.	.	.
B/Brazil/0496/2016	.	.	.	.	.	.	.	.	.	.	.	.	.	V	.	.	.	N	.	.	.	R	.	.	D	.	.	K	.	G	.	.	.
B/Santiago/45998/2016	.	.	.	.	.	.	.	.	.	.	.	.	.	V	.	.	.	N	.	.	.	R	.	.	D	.	.	K	.	G	.	.	.
B/Paraguay/2377/2016	.	.	.	.	.	.	.	.	.	.	.	.	.	V	.	.	.	N	.	.	.	R	.	.	D	.	.	K	.	G	.	.	.
B/Paraguay/5894/2016	.	.	.	.	.	.	.	.	.	F	.	.	.	V	.	.	.	N	.	.	.	R	.	.	D	.	.	K	.	G	.	.	.
B/Santiago/25385/2017	.	.	.	.	.	.	.	.	.	.	.	.	.	V	.	.	.	N	.	.	.	R	.	.	D	.	.	K	.	G	.	.	.
B/Florida/78/2015	.	.	.	.	.	.	.	.	.	F	.	.	.	V	.	.	.	N	.	.	.	R	.	.	D	.	.	K	.	G	T	.	.
B/Argentina/11461/2015	.	.	.	.	.	.	.	.	.	.	.	.	.	V	.	.	.	N	.	.	.	R	.	.	D	.	.	K	.	G	.	.	.
B/Brazil/80287/2015	.	.	.	.	.	.	.	.	.	.	.	.	.	V	.	.	.	N	.	.	.	R	I	.	D	.	.	K	.	G	.	.	.
B/Brazil/4558/2017	.	.	.	.	.	.	.	.	.	.	.	.	.	V	.	.	.	N	.	.	.	R	.	.	D	.	.	K	.	G	.	.	.
B/Santiago/80460/2015	.	.	.	.	.	.	.	.	.	.	.	.	.	V	.	.	.	N	.	.	.	R	.	.	D	.	N	K	.	.	.	.	.
B/Argentina/268/2016	.	.	.	.	.	.	.	.	.	.	.	.	.	V	.	.	.	N	.	.	.	R	.	.	D	.	N	K	.	.	.	.	.
B/Paraguay/8037/2017	.	.	.	.	.	.	.	.	.	.	.	.	.	V	.	.	.	N	.	.	.	R	.	.	D	.	N	K	.	.	.	.	.
**B/Uruguay/289/2015**	-	-	-	K	.	.	.	.	.	.	.	.	.	.	.	.	.	.	.	.	.	R	.	.	D	.	.	K	.	.	.	.	.
**B/Uruguay/345/2015**	-	-	-	.	.	.	.	.	.	.	.	.	.	.	.	.	.	.	.	.	.	R	.	.	D	.	.	K	.	.	.	.	.
**B/Uruguay/335/2015**	-	-	-	-	.	.	.	.	.	.	.	.	.	.	.	.	.	.	.	.	.	R	.	.	D	.	.	K	.	.	.	.	.
**B/Uruguay/02/2016**	T	I	.	.	.	.	.	.	.	.	.	.	.	.	.	.	.	.	.	.	.	R	.	.	D	.	.	K	.	.	.	.	I
**B/Uruguay/98/2015**	T	I	.	.	.	.	.	.	.	.	.	.	.	.	.	.	.	.	.	.	.	R	.	.	D	.	.	K	.	.	.	.	.
**B/Uruguay/102/2015**	T	I	.	.	.	.	.	.	.	.	.	.	.	.	.	.	.	.	.	.	.	R	.	.	D	.	.	K	.	.	.	.	.
**B/Uruguay/342/2015**	-	-	-	.	.	.	.	.	.	.	.	.	.	.	.	.	.	.	.	.	.	R	.	.	D	.	.	K	.	.	.	.	.
B/Brazil/6297/2015	.	.	.	.	.	.	.	.	.	.	.	.	.	.	.	.	.	.	.	.	.	R	.	.	D	.	.	K	.	.	.	.	.
**B/Uruguay/124/2017**	.	.	.	.	.	.	.	.	.	.	.	.	.	.	.	.	.	.	.	.	.	R	.	.	D	.	N	K	.	.	.	.	.
B/Montana/05/2012	.	.	.	.	.	.	.	.	.	F	.	.	.	.	.	.	.	.	.	.	.	R	.	.	D	.	.	K	.	.	.	.	.
B/PuertoMontt/81199/2013	.	.	.	.	.	.	.	.	.	P	.	F	.	.	.	.	.	.	.	.	.	R	.	.	D	.	.	K	.	.	.	.	.
B/Concepcion/264/2014	.	.	.	.	.	.	.	.	.	.	.	.	.	.	.	.	.	.	.	.	.	R	.	.	D	.	.	K	.	.	.	.	.
**B/Uruguay/177/2013**	.	.	.	.	.	.	.	.	.	.	.	.	.	.	.	.	.	.	.	.	.	R	.	.	D	.	.	K	.	.	.	.	
B/Argentina/154/2014	.	.	.	.	.	.	.	.	.	.	.	.	.	.	.	.	.	.	.	.	.	R	.	.	D	.	.	K	.	.	.	.	.
B/BuenosAires/10986152/2014	.	.	.	.	.	.	.	.	.	.	.	.	.	.	.	.	.	.	.	.	.	R	.	.	D	.	.	K	.	.	.	.	.
B/Brazil/2920/2014	.	.	.	.	.	.	.	.	.	.	.	.	.	.	.	.	.	.	E	.	.	R	.	.	D	.	.	K	.	.	.	.	.
B/Santiago/82832/2013	.	.	.	.	.	.	.	.	.	.	.	.	.	.	.	.	.	.	.	.	.	R	.	.	D	.	.	K	.	.	.	.	.
B/Brazil/5137/2014	.	.	.	.	.	.	.	.	.	.	.	.	.	.	.	.	.	.	.	.	.	R	.	.	D	.	.	K	.	.	.	.	.
B/Valparaiso/81181/2015	.	.	.	.	.	.	.	.	.	.	.	.	.	.	.	.	.	.	.	.	.	R	.	K	D	.	.	K	.	.	V	.	.
B/Paraguay/1131/2015	.	.	.	.	.	.	.	.	.	.	.	.	.	.	.	.	.	.	.	.	.	R	.	K	D	.	.	K	.	.	.	.	.
B/Paraguay/4674/2014	.	.	.	.	.	.	.	.	.	.	P	.	.	.	.	.	.	.	.	.	.	R	.	.	D	.	.	K	.	.	.	.	.
B/Paraguay/5133/2015	.	.	.	.	.	.	.	.	.	.	.	.	.	.	.	.	.	.	.	.	.	R	.	.	D	.	.	K	.	.	.	.	.
B/BuenosAires/770/2012	.	.	.	.	.	A	.	.	.	.	.	.	.	.	.	.	.	.	.	.	.	R	.	.	D	.	.	K	.	.	.	.	.
B/Brazil/6004/2013	.	.	.	.	.	A	.	.	.	.	.	.	.	.	.	.	.	.	.	.	.	R	.	.	D	.	.	K	.	.	.	.	.
B/Formosa/V2367/2012	.	.	.	.	.	.	.	.	.	F	.	.	.	.	.	.	.	.	.	.	.	R	.	.	D	.	.	K	.	.	.	.	.
B/Brazil/3742/2013	.	.	.	.	.	.	.	.	.	F	.	.	.	.	.	.	S	.	.	.	.	R	.	.	D	.	N	K	.	.	.	.	.
B/Paraguay/672/2014	.	.	.	.	.	.	.	.	.	.	.	.	.	.	.	.	S	.	.	.	.	R	.	.	D	.	N	K	.	.	.	.	.
**B/Uruguay/406/2014**	-	-	-	.	.	.	.	.	.	F	.	.	.	.	.	.	S	.	.	.	.	R	.	.	D	.	N	K	.	.	.	.	.
**B/Uruguay/578/2014**	-	-	-	.	.	.	.	.	.	F	.	.	.	.	.	.	S	.	.	.	.	R	.	.	D	.	N	K	.	.	.	.	.
B/Santiago/34960/2012	.	.	.	.	.	.	.	.	.	.	.	.	.	.	.	.	.	.	.	.	.	R	.	.	D	.	.	K	.	.	.	.	.
B/Santiago/49303/2012	.	.	.	.	.	.	.	.	.	.	.	.	.	.	.	.	.	.	.	.	.	R	.	.	D	.	.	K	.	.	.	.	.
**B/Uruguay/236/2012**	-	-	-	.	.	.	.	.	.	.	.	.	.	.	.	.	.	.	.	.	.	.	.	.	D	.	.	.	.	.	.	.	.
B/Brazil/0944/2012	.	.	.	.	.	.	.	.	.	.	.	.	.	.	.	.	.	.	.	.	.	.	.	.	D	.	.	.	.	.	.	.	.
B/Paraguay/190/2012	.	.	.	.	.	.	.	.	.	.	.	.	.	.	.	.	.	.	.	.	.	.	.	.	D	.	.	.	.	.	.	.	.
B/Paraguay/252/2012	.	.	.	.	.	.	.	.	.	.	.	.	.	.	.	.	.	.	.	.	.	.	.	.	D	.	.	.	.	.	.	.	.
B/Victoria/304/2006	.	.	.	.	.	.	.	.	.	.	.	.	.	.	.	.	.	.	.	.	.	.	.	.	.	.	.	A	.	.	.	.	.
B/Argentina/R158/2010	.	.	.	.	.	.	.	.	.	.	.	.	I	.	.	.	.	N	.	.	.	.	.	.	.	N	.	A	.	.	V	.	.
B/Uruguay/12/2008	.	.	.	.	.	.	.	.	.	.	.	.	.	.	.	.	.	N	.	.	.	.	.	.	.	.	.	A	.	.	.	.	.
B/Singapore/19/2009	.	.	.	.	.	.	.	G	.	.	.	.	.	.	K	.	.	N	.	.	V	.	.	.	.	.	.	A	.	.	.	.	.
B/Fujian/Gulou1272/2008	.	.	.	.	.	.	.	.	.	F	.	.	.	.	K	.	.	N	.	.	.	.	.	.	.	G	.	A	.	.	.	.	.
B/GuangdongLiwan/1133/2014	.	.	.	.	.	.	.	.	.	.	.	.	.	.	K	.	.	N	.	.	.	.	.	.	.	G	.	A	.	N	.	I	.
B/Victoria/2/87	.	I	S	.	.	.	E	M	.	F	.	.	A	.	K	.	S	N	.	D	.	.	.	.	.	E	R	A	.	.	.	.	.

Table S4. Comparison of the amino acid sequence analysis of the NA of the Influenza B/Victoria viruses from 2012-2019 with B/Brisbane/60/2008 reference strain. A dot (.) denotes same amino acid at the same position in the reference sequence.

	42	45	46	49	55	57	59	60	67	68	73	76	78	88	91	99	106	115	120	125	171	175	186	248	295	320	340	342	343	371	372	373	375	395
B/Phuket/3073/2013	**R**	**V**	**T**	**I**	**A**	**A**	**N**	**V**	**A**	**T**	**P**	**P**	**P**	**P**	**T**	**S**	**T**	**I**	**I**	**K**	**I**	**A**	**R**	**I**	**S**	**K**	**D**	**D**	**E**	**K**	**T**	**K**	**M**	**A**
B/Argentina/13785/2018	.	.	.	M	.	.	.	.	.	.	.	.	.	.	.	.	.	.	.	.	M	.	.	.	.	.	.	K	.	.	N	Q	.	S
**B/Uruguay/1005/2018**	-	-	-	-	-	-	.	.	.	.	.	.	.	.	.	.	.	.	.	.	M	.	.	.	.	.	.	K	.	.	N	Q	.	S
**B/Uruguay/816/2018**	-	-	-	-	-	.	.	.	.	.	.	.	.	.	.	.	.	.	.	.	M	.	.	.	.	.	.	K	.	.	.	Q	.	S
**B/Uruguay/033/2018**				M	.	.	.	.	.	.	.	.	.	.	.	.	.	.	.	.	M	.	.	.	.	.	.	K	.	R	.	Q	.	S
**B/Uruguay/727/2018**	-	-	-	-	-	.	.	.	.	.	.	.	.	.	.	.	.	.	.	.	M	.	.	.	.	.	.	K	.	.	.	Q	.	S
**B/Uruguay/1054/2018**	-	-	-	-	-	.	.	.	.	.	.	.	.	.	.	.	.	.	.	.	M	.	.	.	.	.	.	K	.	.	.	Q	.	S
B/Brazil/7103/2018	.	.	.	M	.	.	.	.	.	.	.	.	.	.	.	.	.	.	.	.	M	.	.	.	.	.	.	K	.	.	.	Q	.	S
B/Santiago/64801/2019	.	.	.	M	.	.	.	.	.	.	.	.	.	.	.	.	.	.	.	.	M	.	.	.	.	.	.	K	.	.	.	Q	.	S
B/Santiago/64840/2019	.	.	.	M	.	.	.	.	.	.	.	.	.	.	.	.	.	.	.	.	M	.	.	.	.	.	.	K	.	.	.	Q	.	S
**B/Uruguay/571/2018**	.	.	.	M	.	.	.	.	.	.	.	.	.	.	.	.	.	.	.	.	M	.	K	.	.	.	.	K	.	.	.	Q	.	.
B/Santiago/75265/2017	.	.	.	M	.	.	.	.	.	.	.	.	.	.	.	.	.	.	.	.	M	.	.	.	.	.	.	K	.	.	.	Q	.	.
B/Santiago/53060/2018	.	.	.	M	.	.	.	.	.	.	.	.	.	.	.	.	.	.	.	.	M	.	.	.	.	.	.	K	.	.	.	Q	.	.
B/Argentina/14/2018	.	.	.	M	.	.	.	.	.	.	.	.	.	.	.	.	.	.	.	.	M	.	.	.	.	.	.	K	.	.	.	Q	.	.
B/Santiago/34517/2018	.	.	.	M	.	.	.	.	.	.	.	.	.	.	.	.	.	.	.	.	M	.	.	.	.	.	.	K	.	.	.	Q	.	.
**B/Uruguay/553/2018**	.	.	.	M	.	.	.	.	.	.	.	Q	.	.	.	.	I	.	.	.	M	.	.	.	.	.	.	K	.	.	.	Q	.	.
**B/Uruguay/1070/2018**	-	-	-	-	-	.	.	.	.	.	.	.	.	.	.	.	I	.	.	.	M	.	.	.	.	.	.	K	.	.	.	Q	.	.
B/Brazil/0379/2018	.	.	I	.	.	.	.	.	.	I	L	.	.	.	.	.	.	.	.	.	.	.	.	.	.	.	.	N	.	.	.	Q	.	.
B/Santiago/81374/2017	.	.	.	.	.	.	.	.	V	.	.	.	.	.	.	.	.	.	.	.	.	.	.	.	.	.	.	N	.	.	.	Q	.	.
B/Paraguay/6288/2018	.	.	.	.	.	.	.	A	V	.	.	.	.	.	.	.	.	.	.	.	.	.	.	.	.	.	.	N	.	.	.	Q	.	.
B/Paraguay/0336/2018	.	.	.	.	.	.	.	A	V	.	.	.	.	.	.	.	.	.	.	.	.	.	.	.	.	.	.	N	.	.	.	Q	.	.
B/Argentina/13252/2017	.	.	.	.	.	.	.	A	V	.	.	.	.	.	.	.	.	.	.	.	.	.	.	.	.	.	.	N	.	.	.	Q	.	.
B/Paraguay/3099/2017	.	.	.	.	.	.	.	A	V	.	.	.	.	.	.	.	.	.	.	.	.	.	.	.	.	.	.	N	.	.	.	Q	.	.
B/Paraguay/8072/2017	.	.	.	.	.	.	.	A	V	.	.	.	.	.	.	.	.	.	.	.	.	.	.	.	.	.	.	N	.	.	.	Q	.	.
B/Argentina/1744/2017	.	.	.	.	.	.	.	A	V	.	.	.	.	.	.	.	.	.	.	.	.	.	.	.	.	.	.	N	.	.	.	Q	.	.
**B/Uruguay/403/2017**	.	.	.	.	.	.	.	A	V	.	.	.	.	.	.	.	.	M	.	.	.	.	.	.	.	.	.	N	.	.	.	Q	.	.
**B/Uruguay/113/2017**	.	.	.	.	.	.	.	A	V	.	.	.	.	.	.	.	.	.	.	.	.	.	.	.	.	.	.	N	.	.	.	Q	.	.
**B/Uruguay/119/2017**	.	.	.	.	.	.	.	A	V	.	.	.	.	.	.	.	.	.	.	.	.	.	.	.	.	.	.	N	.	.	.	Q	.	.
**B/Uruguay/338/2017**	.	.	.	.	.	.	.	A	V	.	.	.	.	.	.	.	.	.	.	.	.	.	.	.	.	.	.	N	.	.	.	Q	.	.
**B/Uruguay/106/2017**	.	.	.	.	.	.	.	A	V	.	.	.	.	.	.	.	.	.	.	.	.	.	.	.	.	.	.	N	.	.	.	Q	.	.
**B/Uruguay/126/2017**	.	.	.	.	.	.	.	A	V	.	.	.	.	.	.	N	.	.	.	.	.	.	.	.	.	.	.	N	.	.	.	Q	.	.
**B/Uruguay/527/2017**	.	.	.	.	.	.	.	A	V	.	.	.	.	.	.	.	.	.	.	.	.	.	.	.	.	.	.	N	.	.	.	Q	.	.
B/Santiago/75552/2015	.	.	.	.	.	.	.	.	.	.	.	.	.	.	.	.	.	.	.	.	.	.	.	.	.	.	.	.	.	.	.	Q	.	.
B/Brazil/8134/2017	.	.	.	.	.	.	.	.	.	A	.	.	.	.	.	.	.	.	.	.	.	.	.	V	R	.	.	N	.	.	.	.	.	.
B/Santiago/20269/2016	.	.	.	.	.	.	.	.	.	A	.	.	.	.	.	.	.	.	.	.	.	.	.	V	R	.	.	N	.	.	.	.	.	.
B/Argentina/220/2016	.	.	.	T	.	.	.	.	.	.	.	.	.	.	.	.	.	.	.	.	.	.	.	V	R	.	.	.	.	.	.	.	.	.
B/Brazil/3889/2017	.	.	.	T	.	.	.	.	.	.	.	.	.	.	.	.	.	.	.	.	.	.	.	.	R	.	.	.	.	.	.	.	.	.
B/Paraguay/5243/2016	.	.	.	T	.	.	.	.	.	.	.	.	.	.	.	.	.	.	.	.	.	.	.	.	R	.	.	.	.	.	.	.	.	.
B/Argentina/11616/2015	.	.	.	.	.	.	.	.	.	.	.	.	.	.	.	.	.	.	.	.	.	.	.	.	.	.	.	N	.	.	.	.	.	.
B/Brazil/0589/2015	.	.	.	.	.	.	.	.	.	.	.	.	.	.	.	.	.	.	.	.	.	.	.	.	.	.	.	N	.	.	.	.	.	.
B/Paraguay/0905/2015	.	.	.	.	.	.	.	.	.	.	.	.	.	.	.	.	.	.	.	.	.	.	.	.	.	.	.	N	.	.	.	.	.	.
B/Valparaiso/49570/2016	.	.	.	.	.	.	.	.	.	.	.	.	.	.	.	.	.	.	V	.	.	.	.	.	.	.	.	N	.	.	.	.	.	.
B/Pernambuco/144718IEC/2016	.	.	.	.	.	.	.	.	.	.	.	.	.	.	.	.	.	.	V	.	.	.	.	.	.	.	.	N	.	.	.	.	.	.
**B/Uruguay/372/2015**	-	-	-	-	-	R	.	.	.	.	.	.	.	.	.	.	.	.	V	.	.	T	.	.	.	.	.	N	.	.	.	.	.	.
B/Pucon/75541/2015	.	.	.	.	.	.	.	.	.	.	.	.	.	.	.	.	.	.	V	.	.	.	.	.	.	.	.	N	.	.	.	.	R	.
B/Paraguay/3296/2016	.	.	.	.	.	.	.	.	.	.	.	.	.	.	.	.	.	.	V	.	.	.	.	.	.	.	.	N	.	.	.	.	.	.
**B/Uruguay/309/2015**	-	-	-	-	-	.	K	F	.	.	.	.	S	.	.	.	.	.	V	.	.	.	.	.	.	.	.	N	.	.	.	.	.	.
B/Brazil/36401/2015	.	.	.	.	.	.	.	.	.	.	.	.	.	.	.	.	.	.	.	.	.	.	.	.	.	.	.	N	.	.	.	.	.	D
B/Brazil/1716/2014	.	.	.	.	.	.	.	.	.	.	.	.	.	.	.	.	.	.	.	.	.	.	.	.	.	.	.	.	.	.	.	.	.	.
B/Brazil/2282/2014	.	.	.	.	.	.	.	.	.	.	.	.	.	.	.	.	.	.	.	.	.	.	.	.	.	.	.	.	.	.	.	.	.	.
B/Santiago/73755/2014	.	.	.	.	.	.	.	.	.	.	.	.	.	.	.	.	.	.	.	.	.	.	.	.	.	.	.	.	.	.	.	.	.	.
B/Santiago/71933/2014	.	.	.	.	.	.	.	.	.	.	.	.	.	.	.	.	.	.	.	.	.	.	.	.	.	.	.	.	.	.	.	.	.	.
**B/Uruguay/143/2014**	.	.	N	.	.	.	.	.	.	.	.	.	.	.	.	.	.	.	.	.	.	.	.	.	.	.	.	.	.	.	.	.	.	.
B/RioGrandeDoNorte/140088/2016	.	.	.	.	.	.	.	.	.	.	.	.	.	.	.	.	.	.	.	.	.	.	.	.	.	.	.	.	.	.	.	.	.	.
**B/Uruguay/422/2014**	-	-	-	-	-	.	.	.	.	.	.	.	.	.	I	.	.	.	.	.	.	.	.	.	.	.	.	.	.	.	.	.	.	.
B/SantaFe/1149/2014	.	.	.	.	.	.	.	.	.	.	.	.	.	.	.	.	.	.	.	.	.	.	.	.	.	.	.	.	.	.	.	.	.	.
B/Argentina/19/2016	.	.	.	.	.	.	.	.	.	.	.	.	.	.	.	.	.	.	.	.	.	.	.	.	.	.	.	.	.	.	.	.	.	.
B/Paraguay/2429/2014	.	.	.	.	.	.	.	.	.	.	.	.	.	.	.	.	.	.	.	.	.	.	.	.	.	.	.	.	.	.	.	.	.	.
B/Paraguay/6712/2015	.	.	.	.	.	.	.	.	.	.	.	.	.	.	.	.	.	M	.	.	.	.	.	.	.	.	.	.	.	.	.	.	.	.
B/Santiago/11896/2012	.	I	.	.	.	.	.	.	.	.	.	.	.	.	.	.	.	.	.	.	.	.	.	.	.	E	N	.	.	.	.	.	.	.
B/Wisconsin/01/2010	.	I	.	.	.	.	.	.	.	.	L	.	.	.	.	.	.	.	.	.	.	.	.	.	.	E	N	.	K	.	.	.	.	.
B/Brazil/325/2013	.	I	.	.	.	.	.	.	.	.	L	.	.	.	.	.	.	.	.	.	.	.	.	.	.	E	N	.	K	.	.	.	.	.
B/Natal/119888/2012	.	I	.	.	.	.	.	.	.	.	L	.	.	.	.	.	.	.	.	.	.	.	.	.	.	E	N	.	K	.	.	.	.	.
B/Santiago/80686/2013	.	I	.	.	.	.	.	.	.	.	F	.	.	.	.	.	.	.	.	.	.	.	.	.	.	E	N	.	K	.	.	.	.	.
B/Paraguay/022/2013	.	I	.	.	.	.	.	.	.	.	L	.	.	.	.	.	.	.	.	.	.	.	.	.	.	E	N	.	K	.	.	.	.	.
B/Paraguay/0068/2014	.	I	.	.	.	.	.	.	.	.	L	.	.	.	.	.	.	.	.	.	.	.	K	.	.	E	N	.	K	.	.	.	.	.
B/Florida/4/2006	Q	I	.	.	.	.	.	.	.	A	L	.	.	.	.	.	.	.	.	T	.	.	K	.	.	E	.	.	K	.	.	.	.	.
B/Santiago/38204/2012	Q	I	.	.	.	.	.	.	.	A	L	.	.	.	.	.	I	.	.	T	.	.	K	V	R	E	.	.	K	.	.	.	.	.
B/Massachusetts/02/2012	Q	I	.	.	.	.	.	.	.	A	L	.	.	.	.	.	I	.	.	T	.	.	K	V	R	E	.	.	K	.	.	.	.	.
B/Brazil/374/2013	Q	I	.	.	T	.	.	.	.	A	L	.	.	.	.	.	I	.	.	T	.	.	K	V	R	E	.	.	K	.	.	.	.	.
B/SantaFe/1079/2014	Q	I	.	.	.	.	.	.	.	A	L	.	.	.	.	.	I	.	.	M	.	.	K	V	R	E	.	.	K	.	.	.	.	V
B/Santiago/23499/2013	Q	I	.	.	.	.	.	.	.	A	L	.	.	.	.	.	I	.	.	T	.	.	K	V	R	E	.	.	K	.	.	.	.	.
**B/Uruguay/252/2012**	-	-	-	-	-	.	.	.	.	A	L	.	.	.	.	.	I	.	.	T	.	.	K	V	R	E	.	.	K	.	.	.	.	.
B/Tucuman/A285/2012	Q	I	.	.	T	.	.	.	.	A	L	.	.	.	.	.	I	.	.	T	.	.	K	V	R	E	.	.	K	.	.	.	.	.
B/BuenosAires/8833/2012	Q	I	.	.	T	.	.	.	.	A	L	.	.	.	.	.	I	.	.	T	.	.	K	V	R	E	.	.	K	.	.	.	.	.
**B/Uruguay/153/2012**	Q	I	.	.	T	.	.	.	.	A	L	.	.	.	.	.	I	.	.	T	.	.	K	V	R	E	.	.	K	.	.	.	.	.
**B/Uruguay/358/2012**	Q	I	.	.	T	.	.	.	.	A	L	.	.	.	.	.	I	.	.	T	.	.	K	V	R	E	.	.	K	.	.	.	.	.
**B/Uruguay/266/2012**	-	-	-	-	-	-	.	.	.	A	L	.	.	.	.	.	I	.	.	T	.	.	K	V	R	E	.	.	K	.	.	.	.	.
**B/Uruguay/515/2012**	-	-	-	-	-	-	.	.	.	A	L	.	.	.	.	.	I	.	.	T	.	T	K	V	R	E	.	.	K	.	.	.	.	.
**B/Uruguay/276/2012**	-	-	-	-	-	-	-	-	.	A	L	.	.	L	.	.	I	.	.	T	.	.	K	V	R	E	.	.	K	.	.	.	.	.
**B/Uruguay/488/2012**	-	-	-	-	-	.	.	.	.	A	L	.	.	.	.	.	I	.	.	T	.	.	K	V	R	E	.	.	K	.	.	.	.	.
B/Yamagata/16/88	P	I	.	K	.	.	.	.	.	.	L	S	.	Q	.	.	.	.	.	.	.	.	.	.	.	E	N	.	K	.	.	E	.	.

Table S5. Comparison of the amino acid sequence analysis of the NA of the Influenza B/Yamagata viruses from 2012-2019 with B/Phuket/3013/2013 reference strain. A dot (.) denotes same amino acid at the same position in the reference sequence.
